# Induced pluripotent stem cells derived from patients carrying mitochondrial mutations exhibit altered bioenergetics and aberrant differentiation potential

**DOI:** 10.1186/s13287-023-03546-7

**Published:** 2023-11-07

**Authors:** Fibi Meshrkey, Kelly M. Scheulin, Christopher M. Littlejohn, Joshua Stabach, Bibhuti Saikia, Vedant Thorat, Yimin Huang, Thomas LaFramboise, Edward J. Lesnefsky, Raj R. Rao, Franklin D. West, Shilpa Iyer

**Affiliations:** 1 Department of Biological Sciences, J. William Fulbright College of Arts and Sciences, University of Arkansas, Science and Engineering 601, Fayetteville, AR 72701 USA; 2 Cell and Molecular Biology Program, University of Arkansas, Fayetteville, AR USA; 3 Department of Histology and Cell Biology, Faculty of Medicine, Alexandria University, Alexandria, Egypt; 4 Regenerative Bioscience Center, University of Georgia, Athens, GA USA; 5 Department of Animal and Dairy Science, University of Georgia, Athens, GA USA; 6 Neuroscience Program, Biomedical and Health Sciences Institute, University of Georgia, Athens, GA USA; 7 Department of Genetics and Genome Sciences, Case Western Reserve University School of Medicine, Cleveland, OH USA; 8 Department of Physiology and Biophysics, Virginia Commonwealth University, Richmond, VA USA; 9 Cardiology Section Medical Service, McGuire Veterans Affairs Medical Center, Richmond, VA USA; 10 Department of Biochemistry and Molecular Biology, Virginia Commonwealth University, Richmond, VA USA; 11 Division of Cardiology, Department of Internal Medicine, Pauley Heart Center, Virginia Commonwealth University, Richmond, VA USA; 12 Department of Biomedical Engineering, College of Engineering, University of Arkansas, Fayetteville, AR USA

**Keywords:** Mitochondrial disease, hiPSC, Pluripotent stem cell, Reprogramming, Bioenergetics, Respiration, Differentiation

## Abstract

**Background:**

Human mitochondrial DNA mutations are associated with common to rare mitochondrial disorders, which are multisystemic with complex clinical pathologies. The pathologies of these diseases are poorly understood and have no FDA-approved treatments leading to symptom management. Leigh syndrome (LS) is a pediatric mitochondrial disorder that affects the central nervous system during early development and causes death in infancy. Since there are no adequate models for understanding the rapid fatality associated with LS, human-induced pluripotent stem cell (hiPSC) technology has been recognized as a useful approach to generate patient-specific stem cells for disease modeling and understanding the origins of the phenotype.

**Methods:**

hiPSCs were generated from control BJ and four disease fibroblast lines using a cocktail of non-modified reprogramming and immune evasion mRNAs and microRNAs. Expression of hiPSC-associated intracellular and cell surface markers was identified by immunofluorescence and flow cytometry. Karyotyping of hiPSCs was performed with cytogenetic analysis. Sanger and next-generation sequencing were used to detect and quantify the mutation in all hiPSCs. The mitochondrial respiration ability and glycolytic function were measured by the Seahorse Bioscience XFe96 extracellular flux analyzer.

**Results:**

Reprogrammed hiPSCs expressed pluripotent stem cell markers including transcription factors POU5F1, NANOG and SOX2 and cell surface markers SSEA4, TRA-1-60 and TRA-1-81 at the protein level. Sanger sequencing analysis confirmed the presence of mutations in all reprogrammed hiPSCs. Next-generation sequencing demonstrated the variable presence of mutant mtDNA in reprogrammed hiPSCs. Cytogenetic analyses confirmed the presence of normal karyotype in all reprogrammed hiPSCs. Patient-derived hiPSCs demonstrated decreased maximal mitochondrial respiration, while mitochondrial ATP production was not significantly different between the control and disease hiPSCs. In line with low maximal respiration, the spare respiratory capacity was lower in all the disease hiPSCs. The hiPSCs also demonstrated neural and cardiac differentiation potential.

**Conclusion:**

Overall, the hiPSCs exhibited variable mitochondrial dysfunction that may alter their differentiation potential and provide key insights into clinically relevant developmental perturbations.

**Supplementary Information:**

The online version contains supplementary material available at 10.1186/s13287-023-03546-7.

## Introduction

Mutations in mitochondrial DNA (mtDNA) are associated with a wide range of human diseases with complex pathologies such as developmental delays, brain damage, cardiomyopathy, lactic acidosis, autism and infertility [1, 2]. The molecular mechanisms underlying disease pathology of childhood mitochondrial diseases during early human development are poorly understood, with a specific need to address the impact of pathogenic mtDNA mutations on mitochondrial bioenergetics. Mutations may arise from aberrant repair of DNA damaged by oxidative stress and have been found in tissues requiring high energy demands including the brain, skeletal muscle and heart. Initially, when a mutation occurs, cells contain mixtures of wild type and mutant mtDNA, a phenomenon referred to as heteroplasmy. Heteroplasmy can occur at both the cellular and tissue levels and is capable of shifting the mtDNA genotype of the daughter cells by a process of replicative segregation [3–5]. Cells and tissues that are metabolically active are highly sensitive, however less tolerant to the deleterious effects of mtDNA mutations.

Mitochondria contribute an essential role in maintaining metabolic homeostasis by producing energy in the form of adenosine triphosphate (ATP) [6–8]. They also serve as the hub for cellular activities including lipid metabolism, the citric acid cycle and oxidative phosphorylation (OXPHOS) [6]. In the inner mitochondrial membrane, electrons are transferred via the electron transport chain (ETC) within Complexes I–IV to produce O_2_ and H_2_O. The electron transport results in subsequent translocation of protons from the matrix into the intermembrane space, creating a proton gradient in combination with the inward-negative mitochondrial membrane potential to drive the molecular motor, ATP synthase (Complex V), to synthesize ATP. An impairment in the ETC or within the assembly of any of the complexes results in metabolic malfunction in cells and tissues. The ETC is a vast complex containing ~ 90 different subunits, which comprise the five enzyme complexes [9, 10]. Of these subunits, mtDNA encodes 13 subunits while nuclear DNA (nDNA) encodes ~ 77 subunits [10]. Together, the nDNA and mtDNA coordinate the synthesis of subunits that come together to form the individual complexes that compose the ETC, subsequently allowing the mitochondria to function as the core energy producer for cellular needs. Consequently, an error in nDNA or mtDNA encoding proteins that make up any of the subunits could result in inborn errors of metabolism leading to malfunction of the many cellular processes requiring efficient functioning of the ETC. Examples of mtDNA mutations that impact Complex I function of the ETC include 10158 T > C-present in the *MTND3* gene [11] and 12706 T > C-present in the *MTND5* gene [12], while those that impact Complex V function include 8993 T > G and 9185 T > C-present in the *MTATP6* gene [13–15]. Numerous studies have demonstrated the potential for these point mutations to not only alter specific ETC proteins, but also the structure and entire multiprotein assembly, thus leading to bioenergetic and cellular dysfunction [13–16].

Many mitochondrial disorders arise due to specific mtDNA mutations leading to a variety of conditions affecting the brain, eyes, heart, muscle, liver, kidney and gastrointestinal tract [1, 2, 4, 5, 14, 17–19]. These disorders can also affect the whole organ or a tissue and disrupt numerous metabolic pathways within the mitochondria, thus making them particularly vulnerable to insults to the mitochondrial genome. Among the inherited metabolic diseases, those that affect the mitochondria frequently arise early in development and cause fatality in infants [20]. A classic mitochondrial disorder is Leigh syndrome (LS) which affects mental and motor activity, wherein disease severity and developmental delay result in fatality from respiratory failure early in life [17, 21]. Although many mtDNA mutations (8993 T > G and 9185 T > C) have been implicated in LS, the genetic basis and the biochemical consequences are currently unknown in early development. Clinical symptoms include neurodevelopmental deterioration, which is often accompanied by brainstem dysfunction [22]. While the clinical presentations might differ between individuals, LS symptoms largely represent the areas in the brain (brainstem, cerebellum, basal ganglia, oculomotor and cranial nerves) involved in its pathology [23]. Besides neurodevelopmental problems, many studies have also revealed the presence of cardiac defects like hypertrophic and dilated cardiomyopathy [24–27]. Like most other mitochondrial diseases, LS is clinically and genetically heterogeneous, resulting in a diverse phenotypic spectrum. The heterogeneous nature of LS can also be attributed in part to the complex nature of the mitochondrial ETC, which is composed of subunits that are encoded by both nuclear (nDNA) and mitochondrial DNA (mtDNA) [8, 22, 27–31] with mutations in either genomes coding for different ETC subunits resulting in LS. There is thus growing interest in studying such rare mitochondrial disorders like LS, because they may shed light into events that occur during aging and provide insight into the causes and consequences of energy dysfunction that occur in the more common age-associated diseases that impact mitochondrial function in the brain, heart and muscle. Therefore, the creation of patient-specific human-induced pluripotent stem cells (hiPSCs) containing defined mutant mtDNA related to mitochondrial disorders is worthy of further study as it will provide key insights into the role of the mitochondrial genome and accompanying bioenergetic dysfunction in early embryonic development and disease progression.

LS-hiPSC reprogramed from patient fibroblasts that are considered equivalent to the inner cell mass of the blastocyst-stage embryo have been recently generated by our team [32] and others [33–37]. These LS-hiPSCs possess dual properties of unlimited self-renewal and the pluripotent potential to differentiate into the three germ layers to form different tissues and can serve as relevant models to understand early human development [35] and mitochondrial diseases [32, 38, 39]. Specifically, we have demonstrated the ability to use non-viral approaches to reprogram patient fibroblasts to generate LS-hiPSCs that continue to exhibit the mtDNA mutation, while maintaining differentiation potential [32], while other studies demonstrated that isogenic hiPSCs generated using viral approaches exhibited variable mutant mtDNA levels, which influenced the cell fate and mitochondrial function [38, 39]. Despite these advances, many questions remain including the types of functional deficits caused by different LS mutations in mtDNA.

In this study, we have used a cocktail of non-modified reprogramming and immune evasion mRNAs and microRNAs to generate multiple hiPSCs from patient fibroblast cells that contain point mutations impacting Complex I and Complex V function. We fully characterized the generated hiPSCs using immunocytochemical and flow cytometry approaches, cytogenetic analyses to demonstrate normal karyotype, and Sanger and next-generation sequencing to detect and quantify mutation burden and thoroughly assessed the mitochondrial respiration and glycolytic function to study the biochemical consequences of mitochondrial genome perturbations during early development. 

## Materials and methods

### Cell culture

Cultures of healthy control BJ (ATCC® CRL-2522™) fibroblasts (ATCC, Manassas, VA) and four patient-derived diseased fibroblasts (MT-ATP6-fbSBG1, MT-ATP6-fbSBG2, MT-ATP6-fbSBG3 and MT-ND5-fbSBG5) were obtained from the Medical University of Salzburg, Austria. All patients carried inherited pathogenic point mutations in mtDNA. All of the mitochondrial disease subjects selected for this study were pediatric patients exhibiting a range of clinical symptoms from mild myopathy to LS to severe neonatal lactic acidosis [28, 29, 40]. The clinical information of the patients from whom fibroblasts were obtained and used in this study is detailed in Table 1. These cells were maintained in fibroblast expansion medium that consisted of minimal essential medium (MEM) (Thermo Fisher Scientific, Waltham, MA), 10% fetal bovine serum (FBS) (GE Healthcare—HyClone™, Chicago, IL) and 2 mM l-glutamine. Fibroblasts were enzymatically passaged in 0.05% Trypsin–EDTA (Thermo Fisher Scientific).

**Table 1:** Clinical Information of patient fibroblast hiPSC lines

Sample name	Mutation	Gene	Clinical information	Age at diagnosis	Sex
BJ-hiPSC	–	–	Healthy control	Neonate	M
SBG1-hiPSC	8993 T > G	*MTATP6*	Morbus leigh	3 years	F
SBG2-hiPSC	8993 T > G	*MTATP6*	Developmental delay, abnormal gait	4 years	M
SBG3-hiPSC	9185 T > C	*MTATP6*	Myopathy	23 years	F
SBG5-hiPSC	12706 T > C	*MTND5*	Severe neonatal lactic acidosis	8 months	F

Once reprogrammed, hiPSCs were maintained in NutriStem hPSC xeno-free (XF) medium (Biological Industries, Cromwell, CT) with Stemolecule Y27632 Dihydrochloride Hydrate (Reprocell, Beltsville, MD) on a highly purified and refined laminin-511 E8 fragment matrix, iMatrix 511(Reprocell) on a 24-h feeding schedule. hiPSCs were enzymatically passaged once reaching 70–80% confluency at a split ratio of 1:3 using StemPro® Accutase® (Thermo Fisher Scientific). All cell cultures were maintained without the use of antibiotics, handled in Biosafety Type II sterile hoods regularly cleaned with UV irradiation and 70% ethanol and grown in 37 °C incubators at 5% CO_2_ and 95% humidity.

### Somatic cell reprogramming to hiPSCs using a cocktail of non-modified reprogramming and immune evasion RNAs

**Fig. 1:**
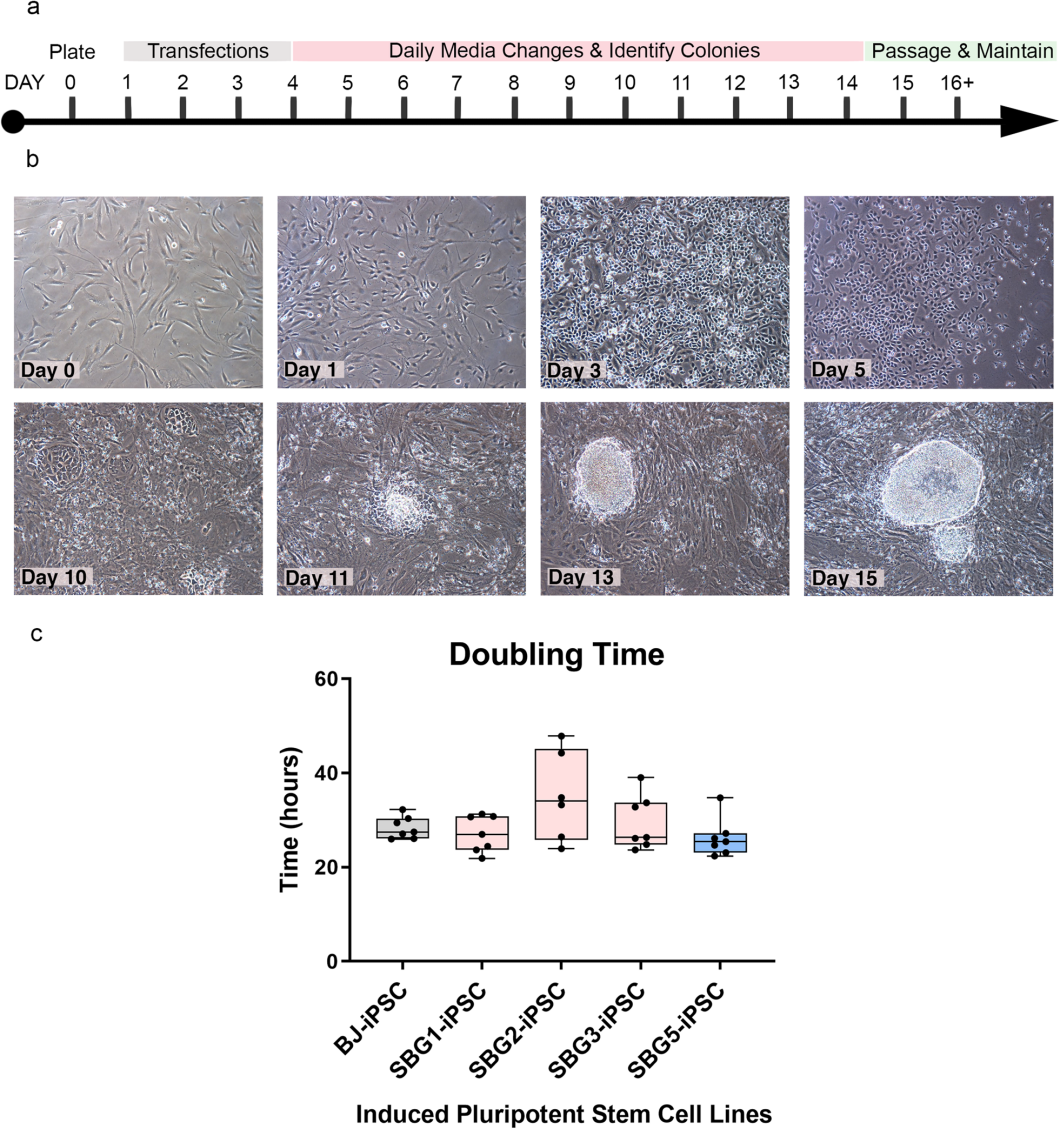
Human patient dermal fibroblasts reprogrammed into hiPSCs using mRNA–microRNA approach. **a** Reprogramming schematic for mRNA and microRNA transfections. **b** Bright-field images (10×) of human disease fibroblasts undergoing reprogramming. Day 0 fibroblasts displayed flat and elongated morphology typical of fibroblasts. Days 3 transfected cells show morphological changes including a rounded morphology and apoptosis, which are consistent with the reprogramming process. Days 10–15 cultures show colony formation, a key iPSC characteristic when cells are in contact with fibroblasts. Multipolar spindle-shaped fibroblast cells transitioning to a more compact cobblestone appearance at day 10, with early stem cell colonies arising by day 11–13 and expanding into well-circumscribed pluripotent stem cell colonies at day 15. Isolated stem cells maintain characteristic well-circumscribed morphology with minimal spontaneous differentiations through 20 + passages. iPSC, induced pluripotent stem cell; **c** Doubling time analyses indicate that all hiPSCs exhibit similar proliferation times representative of actively dividing cells

Putative hiPSCs were generated using the StemRNA 3rd Generation Reprogramming Kit for Adult and Neonatal Human Fibroblasts (Reprocell). Briefly, 1 × 10^5^ fibroblasts were plated in 35-mm dishes in fibroblast expansion medium on iMatrix extracellular substrate at day 0. On day 1, culture medium was switched to NutriStem and a non-modified RNA-reprogramming cocktail was added to the culture for four days of overnight transfections (Fig. 1). The non-modified RNA-reprogramming cocktail consisted of non-modified microRNAs, reprogramming factors (POU5F1 (aka OCT4), SOX2, Klf4, cMyc, Nanog and Lin28) and immune evasion factors (E3, K3 and B18). Daily medium changes were performed until colonies were large enough to be isolated. At day 13–18, putative hiPSCs were identified and isolated by enzymatically passaged with StemPro® Accutase® (Thermo Fisher Scientific) and maintained in culture for over 4–9 passages until apoptosis decreased. Select disease cell lines (SBG1, SBG3) required multiple attempts in the reprogramming process. The newly created hiPSCs were labeled (*MT-ATP6*-SBG1-hiPSC1, *MT-ATP6*-SBG2-hiPSC2, *MT-ATP6*-SBG3-hiPSC3 and *MT-ND5*-SBG5-hiPSC5).

Doubling time assay was performed using hiPSCs between passages 11 and 12 by manual counts (*n* = 3) at 12, 24, 36 and 48 h after plating. Population doubling time was determined using an exponential regression curve fitting (https://www.doubling-time.com/compute_more.php).

### Immunocytochemical analysis

For immunocytochemical detection of the pluripotency markers POU5F1, SOX2, SSEA4, TRA-1-60 and TRA-1-81, cells were cultured in NutriStem medium on iMatrix-coated 4 well Permanox® slides (Nunc Lab-Tek® Chamber Slide™ System; Thermo Fisher Scientific) [41]. Cells were fixed with 4% paraformaldehyde (PFA) solution. For intracellular epitope antibody staining, fixed cells were permeabilized with blocking solution containing 0.3% Triton X-100, 1% polyvinylpyrrolidone and 3% chicken serum in PBS with Ca^2+^ and Mg^2+^. Intracellular staining utilized a PBS with Ca^2+^ and Mg^2+^ wash buffer containing 0.05% Tween 20. For extracellular epitopes, fixed cells were blocked in PBS with Ca^2+^ and Mg^2+^ containing 6% chicken serum and washed with PBS with Ca^2+^ and Mg^2+^. Primary antibodies used for immunocytochemical hiPSC pluripotency characterization were POU5F1/OCT4 (Santa Cruz sc-5279 (mouse) or sc-9081 (rabbit); 1:200), SOX2 (R&D Systems MAB2018; 1:200), SSEA4 (Invitrogen MA1-021; 1:50), TRA-1-60 (Stemgent 09-0068; 1:50), TRA-1-81 (Stemgent 09-0069; 1:50). Primary antibodies were detected by fluorophore-labeled secondary antibodies Alexa Fluor 488 (Invitrogen; 1:1000) and Alexa Fluor 594 (Invitrogen; 1:1000). Nuclei were stained and slides were mounted with Prolong Gold (Invitrogen) with 4′6-diamidino-2-phenylindole (DAPI) (1:1000). Slides were images on a Zeiss LSM 880 confocal microscope.

### Flow cytometry analysis

For flow cytometry analysis of multiple pluripotency markers, cells were fixed in 4% PFA and blocked in PBS without Ca^2+^ and Mg^2+^ in 6% chicken serum. For intracellular marker detection, cells were permeabilized 3 times by adding 0.05% Tween 20 in block solution. Cells were blocked for 45 min at room temperature. Primary antibodies against POU5F1/OCT4 (Santa Cruz sc-5279; 1:200), SOX2 (R&D Systems MAB2018; 1:200), Nanog (Invitrogen PA1-097; 1:200), SSEA4 (Invitrogen MA1-021; 1:50), TRA-1-60 (Stemgent 09-0068; 1:50) and TRA-1-81 (Stemgent 09-0069; 1:50) were used for flow cytometry. Primary antibodies were detected using fluorescently conjugated secondary antibody Alexa Fluor 488 (Invitrogen; 1:000) and Alexa Fluor 594 (Invitrogen; 1:000). Cells were analyzed using the Quanteon analyzer (Agilent, Santa Clara, CA) and FlowJo cytometry analysis software (Tree Star).

### Cytogenetic analysis

Upon reaching a 50–60% confluence, cell cultures were treated with KaryoMAX colcemid (Thermo Fisher Scientific) at a final concentration of 0.1 μg/ml for 40 min. Cells were subsequently enzymatically detached using StemPro® Accutase® (Thermo Fisher Scientific) with Accutase inactivation achieved through resuspension in Nutristem medium. Cells were then spun for ten minutes at 500 g and resuspended in a heated hypotonic solution of 0.075 M KCl for 30 min followed by fixation with 3:1 methanol to glacial acetic acid. Fixed cells were stored at 4 °C for at least 24 h before being resuspended in 0.5 ml of 3:1 methanol to glacial acetic acid fixative. Cells were dropped onto slides and dried over a humidity zone created using heat block with layer damp paper towel. Slides were then aged for 24 h in a 55 °C oven. GTG banding patterns were produced using pancreatin and Wright’s stain with FBS being used to inactivate the pancreatin. Images were taken using a 100 × objective mounted on an Evos FL inverted microscope. Karyotypes were produced from images using the SmartType software platform.

### DNA isolation and purification

Frozen cell pellets containing ~ 2 × 10^6^ cells were thawed and processed. The QIAamp DNA mini kit (Qiagen, Valencia, CA, USA) manufacturer protocol was followed to extract total DNA, which resulted in an elution of 100 μl of distilled water (dH_2_O) and total DNA from all cells. The 100 μl solutions containing the genomic DNA were further treated with 1 μl of RNaseA for 1 h at 37 °C to avoid RNA contamination. The gDNA was quantified using DeNovix UV/Vis Spectrophotometer (DeNovix Inc. Wilmington, DE, USA). A blank of 1.0 μl of dH_2_O was used to establish a zero, and 1.0 μl of each sample was used to determine the concentration.

### PCR and sanger sequencing analysis

Primers for use in PCR were generated using the human mtDNA sequence provided by mitomap.org/MITOMAP and IDT’s Primer Quest tool (IDT, Coralville, Iowa) and Primer 3 (https://bioinfo.ut.ee/primer3/). A standard PCR was carried out using the Takara Taq PCR Amplification Kit (Clontech Mountain View, CA, USA). The targeted gene region was amplified using the primer pairs shown in Additional file 1: Table S1.PCR was performed in a total volume of 50 μl, containing 25 μl, master mix (Promega, Madison, WI, USA), 0.2 μmol of each primer and 100–400 ng of genomic gDNA.

Thermal cycling conditions were 5 min at 95 °C, followed by 36 cycles [30 s at 95 °C, 30 s at the annealing temperature of the primers (52–65 °C) and 30 s at 72 °C] and a final extension for 5 min at 72 °C and a hold at 4 °C for infinity. After PCR reactions, the PCR products were run on 2% agarose gel and DNA band was excised. Further, the gel purification was carried out using QIAEX II Gel extraction Kit (Qiagen, LLC, USA). The DNA was quantified using DENOVIX, spectrophotometer. Finally, the purified PCR products were shipped for Sanger sequencing to UAMS sequencing core. Samples were sequenced at the UAMS Sequencing Core Facility using a 3500 Genetic Analyzer (Applied Biosystems, Foster City, CA). The genotyping and fragment sizing were done on a 3130XL (Applied Biosystems). The files generated from the sequencing were observed using CodonCode Aligner (CodonCode Corporation, Centerville, MA).

### Next-generation sequencing for heteroplasmy analysis

The DNA concentration was verified using a Qubit fluorometer (Thermo Scientific). Instead of the standard DNA fragmentation, an enzymatic fragmentation was performed using the KAPA Frag Enzyme from the KAPA HyperPlus Library Preparation Kit (KAPA Biosystems, Wilmington, MA). This alternative was performed to increase yield during the fragmentation step. Fragmented DNA was purified using Ampure beads (Beckman Coulter, Brea, CA). DNA libraries were prepared using the Accel-NGS 2S Plus DNA Library Kit (Swift Biosciences, Ann Arbor, MI). Ten PCR cycles were carried out during the Library Amplification step. The final libraries were analyzed with a 2100 Bioanalyzer to assess library size distribution (Agilent Technologies, Santa Clara, CA). DNA libraries were quantified with the KAPA Library Quantification Kit to ensure accuracy (KAPA Biosystems). Based on the qPCR results, the DNA libraries were compiled in equimolar amounts and sequenced with the HiSeq 2500 using TruSeq v3 reagents according to the 2 × 100 bp protocol (Illumina, San Diego, CA).

Heteroplasmy levels were extracted from the FASTQ files as previously described [42]. Briefly, we first filtered out reads that were likely to be nuclear mitochondrial sequences (NuMTs). NuMTs are DNA sequences that are harbored in the nuclear genome, but closely match sequence in the mitochondrial genome [43]. Reads that aligned, using Burrows-Wheeler Alignment Tool [44], with up to one mismatch to the nuclear reference sequence GRCh38 (having removed the mitochondrial revised Cambridge reference sequence (rCRS) [45] were excluded from downstream analysis. Resulting reads were realigned to rCRS and read counts of the mutant and wild-type alleles were extracted using SAMtools mpileup [46]. From these counts, the mutant heteroplasmy level was computed as: (mutant allele counts)/(total counts).

### Mitochondrial oxygen consumption detection and glycolysis function analysis

Although metabolic shifting is essential for successful reprogramming, mitochondria still play an important role in regulating the fate of hiPSCs [47–51]. In this study, we evaluated the metabolic state in the generated hiPSCs to further understand the influence of mitochondrial genome perturbations on cellular bioenergetics. Changes in oxygen consumption were measured in real time using XFe96 extracellular flux analyzer. Seahorse XFe96 Cell Mito Stress Test Kit and glycolytic rate assay kit (Seahorse Biosciences, USA) were used as per manufacturer’s instructions. Corning® Cell-Tak™ Cell and Tissue Adhesive (Corning, NY, USA) was used to immobilize the cells (following manufacturer’s procedure) for non-adherent cells. Prior to use in XFe96, hiPSCs were detached using Accutase and seeded into the coated plate with a previously optimized number of 25,000 cells per well. The experiments were conducted on three different days under the same conditions between control and diseased cell lines which have been designated as the three biological replicates. One each day, and for each cell line, 8–12 technical replicates were used to measure mitochondrial respiration and glycolytic profile assays. Statistical significance has been conducted between each patient cell line to the control cell line between the three biological replicates.

The cells were supplemented with 180 µl Mito-stress complete seahorse medium, after which the cells were incubated in a non-CO_2_ incubator at 37^ °^C for one hour. Respiration was measured using the classic mitochondrial inhibitors, specific for complex I and III subunits, such as Rotenone and Antimycin A (0.5 μM final concentrations each). Maximum respiration was measured by addition of an uncoupler FCCP (1.0 μM final concentration) and Oligomycin (1.0 μM final concentration) was added to measure proton leak. The readouts were normalized to cell number and analyzed using Seahorse XF96 Wave software.

Many studies have shown that hiPSCs and human embryonic stem cells (hESCs) use the glycolytic pathway to maintain pluripotency [52]. Given that these new lines contained point mutations in the *mt-ND5* genes in Complex I and *mt-ATP6* genes in Complex V of the ETC, we hypothesized that these cell lines would exhibit glycolytic defects early in development. Therefore, a glycolytic rate assay using the XFe96 based on the following procedure was performed. First, cells were cultured in buffered (5 mM HEPES buffer) Seahorse medium supplemented with glucose and pyruvate and the proton efflux rate (PER) was measured. Then, rotenone and antimycin A were added to inhibit mitochondrial-derived proton efflux. Finally, 2-DG was added to inhibit glycolysis. The different assay parameters: basal glycolysis, compensatory glycolysis, total proton efflux and post-2-DG acidification, were normalized to cell number and analyzed using Seahorse XFe96 Wave software.

### Differentiation into neural and cardiac lineages

iPSC underwent neural differentiation utilizing previously published protocols [53–55]. Briefly, hiPSCs were grown on iMatrix (Amsbio) coated plates and cultured in Nutristem XF (Sartorius) xeno-free, serum-free hiPSC growth medium until reaching > 80% confluency. Nutristem XF medium was removed and replaced with neural induction medium consisting of Essential 6TM medium (E6, Invitrogen) supplied with 100 nM LDN193189 (Selleckchem) and 10 μM SB431542 (Selleckchem). Medium was changed daily. After day 7, observed neural rosettes were manually collected using a glass hook under a dissection scope. iPSC-derived rosettes were cultured on iMatrix in neural expansion medium consisting of KnockOutTM DMEM/F-12 (Life Technologies), StemPro Neural Supplement (50×, ThermoFisher Scientific), 20 ng/ml bFGFs (Peprotech), 10 ng/ml EGFs (Peprotech), Glutamax I (100×, Gibco) and 2 μg/ml heparin (Sigma). Medium was changed daily, and cells were passaged using Accutase every 3 days.

Induced PSCs were differentiated into CMs in monolayer culture systems utilizing previously published protocols [56, 57]. Briefly, hiPSCs were cultured in Nutristem (Reprocell, Beltsville, MD) on iMatrix (Reprocell, Beltsville, MD) coated dishes and passaged when they reach 80% confluency using Accutase (Thermo Fisher Scientific, Waltham, MA). For differentiation, cells were seeded at a density of 8 × 10^5^ cells/well in iMatrix-coated 12-well plates (VWR, Radnor, PA) in Nutristem medium. Three days after the cell seeding, differentiation was induced by replacing the maintenance medium with RPMI 1640 medium (Thermo Fisher Scientific, Waltham, MA) supplemented with 213 μg/ml ascorbic acid (Acros Organics, Thermo Fisher Scientific, Waltham, MA) and 6 μM CHIR99021 (Selleckchem, Houston, TX). Twenty-four hours later, the medium was replaced by RPMI supplemented with 213 μg/ml ascorbic acid. Seventy-two hours later, half of the medium was changed with RPMI supplemented with ascorbic acid, and 6 μM IWP-2 (Tocris Bioscience, Ellisville, MO). On day 5 of differentiation induction, the medium was replaced by RPMI supplemented with the ascorbic acid. From day 7 and every 3 days, cells were maintained with RPMI supplemented with 2% B27 Supplement (Thermo Fisher Scientific, Waltham, MA) and ascorbic acid. Cells were maintained under humidified atmosphere with 5% CO_2_, 37 °C. Beating started around day 8–12 and beating videos were recorded using digital Nikon camera (CoolPixP520, Melville, NY).

The detection of CMs markers was performed using previously published immunocytochemical methods [57]. Cells were passaged in four well staining chambers (Thermo Fisher Scientific, Waltham, MA) and cultured in RPMI supplemented with B27 Supplement and ascorbic acid for 3–4 days. Cells were then fixed and permeabilized using acetone (Sigma-Aldrich, St. Louis, MO)/methanol (EMD Millipore, Billerica, Massachusetts) mixture (1:1) for 20 min at − 20 °C. Cells were washed with 1 × DPBS (Corning, Glendale, AZ) and blocked in 2% bovine serum albumin (BSA, Sigma-Aldrich, St. Louis, MO) for 60 min at room temperature (R.T.). Cells were then incubated with primary antibodies diluted in 2% BSA at R.T. for 1 h. Antibodies used are monoclonal α-sarcomeric actinin (1:100, Sigma-Aldrich, St. Louis, MO) and recombinant anti-cardiac Troponin T antibody [EPR20266] (1:150, Abcam, Waltham, MA). Cells were then washed three times in 1 × DPBS and incubated with secondary antibodies either goat antirabbit 594 (1:500, Thermo Fisher Scientific, Waltham, MA) or goat antimouse 488 (1:500, Thermo Fisher Scientific, Waltham, MA) for 1 h. at R.T. After three washing steps in PBS, cell nuclei were counterstained and coated with ProLong™ Gold Antifade Mountant with DAPI (Thermo Fisher Scientific, Waltham, MA). Samples were visualized through EVOS FL inverted light/epifluorescence microscope with 40×/0.65 objective and a Sony ICX445 monochrome CCD digital camera. Red fluorescence representing cardio-troponin was measured using a 530 nm excitation and a 593 nm emission filter set. Green fluorescence representing α-sarcomeric actinin was measured using a 470 nm excitation and a 525 nm emission filter set. Blue fluorescence representing the nucleus was measured using a 360 nm excitation and a 447 nm emission filter set. Beating frequency was measured as previously reported [58]. The obtained CMs were visualized using EVOS FL inverted light/epifluorescence microscope with 20×/0.45 objective and a Sony ICX445 monochrome CCD digital camera mounted on the microscope. To assess the beating rate in the control and the diseased—hiPSCs derived CMs, videos of the spontaneously beating area of CMs were recorded using an external digital Nikon camera. CM videos were recorded for each cell line for at least 1 min each from different beating areas of two independent differentiation experiments. Beating analysis of the obtained videos was achieved by counting the beats rate for 1 min in the acquired videos using a manual counter and a timer.

### Statistical analysis

In order to ensure scientific rigor and reproducibility, for the bioenergetics analyses, an ANOVA design accounting for 3 biological and 8–12 technical replicates from control (BJ-hiPSC) and diseased (SBG1, SBG2, SBG3, SBG5 hiPSCs) that are nested within groups was used to identify any differences with respect to control BJ-hiPSCs. Post-hoc Tukey HSD tests were used to identify differences among specific groups. An unpaired *t*-test was applied when comparing between two groups. Data are presented as the mean ± standard deviation (SD) and were analyzed using the GraphPad Prism 7 program (GraphPad Software, San Diego, CA, USA). A *p* < 0.05 was considered significant.

## Results

### hiPSCs are generated from reprogrammed patient fibroblasts

To investigate the ability of patient fibroblast cells with single-point mutations in the mitochondrial genome to undergo iPSC reprogramming using an RNA approach, SBG1-3,5 and BJ fibroblast cell lines were transfected with non-modified POU5F1, SOX2, KLF4, cMYC, NANOG and LIN28 mRNAs, immune evasion E3, K3 and B18 mRNAs, and reprogramming-enhanced mature, double-stranded microRNAs from the 302/367 cluster. Fibroblasts were transfected daily for four days in feeder-free and xeno-free culture conditions to prevent contamination from non-human cells and biological material. After 10–11 days, small rounded, putative hiPSC colonies began to arise that developed into well-circumscribed, compact colonies by day 15 (Fig. 1). Patient cell lines did show variability in their ability to be successfully reprogrammed with *mtATP6*-SBG2 and *mtND5-*SBG5 lines successfully reprogrammed during the first attempt to generate stable hiPSC lines, comparable to control BJ line. The SBG1 line underwent the reprogramming process twice before forming stable putative hiPSC lines, and the SBG3 line underwent three rounds of reprogramming to generate stable lines. Once reprogrammed, the respective hiPSC lines were capable of robust expansion with comparable proliferation rates (SBG1-hiPSC1—27.10 h population doubling time; SBG2-hiPSC2—38.33 h; SBG3-hiPSC3—29.49 h; SBG5-hiPSC5—26.23 h) to CTL-BJ-hiPSC control cell line (28.37 h). After stable proliferation and 12 + passages, these stem cell lines were assessed for pluripotency characteristics.

### hiPSCs express markers characteristic of pluripotent stem cells

**Fig. 2:**
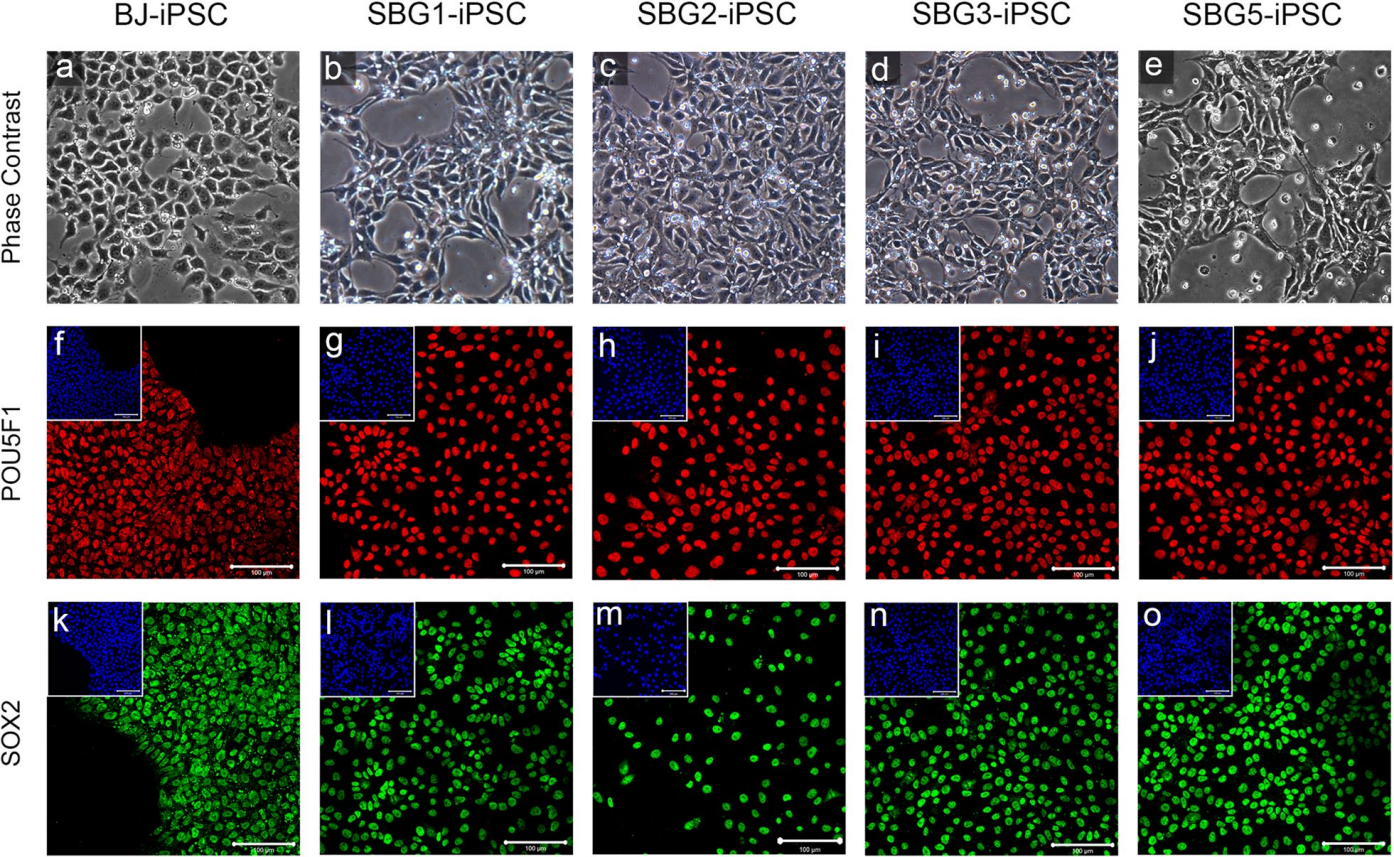
hiPSCs display classical pluripotent stem cell morphology and positively express core pluripotency transcription factors. Phase contrast images of healthy control **a** BJ-hiPSC and diseased **b**–**e** SBG1, 2, 3, 5 hiPSCs show the presence of pluripotent stem cell morphology with cells exhibiting high nuclear-cytoplasmic ratio and large nucleoli, representative of actively dividing undifferentiated cells. Healthy control BJ-hiPSC (**f**, **k**) and diseased SBG1-hiPSC (**g**, **l**), SBG2-hiPSC (h,m), SBG3-hiPSC (**i**, **n**) and SBG5-hiPSC **j**, **o** are positive for POU5F1 (**f**–**j**) and SOX2 (**k**–**o** pluripotent markers. Image inserts contain corresponding DAPI nuclear counterstain. DAPI, 4’,6-diamidino-2-phenylindole. Scale bar = 100 µm

**Fig. 3:**
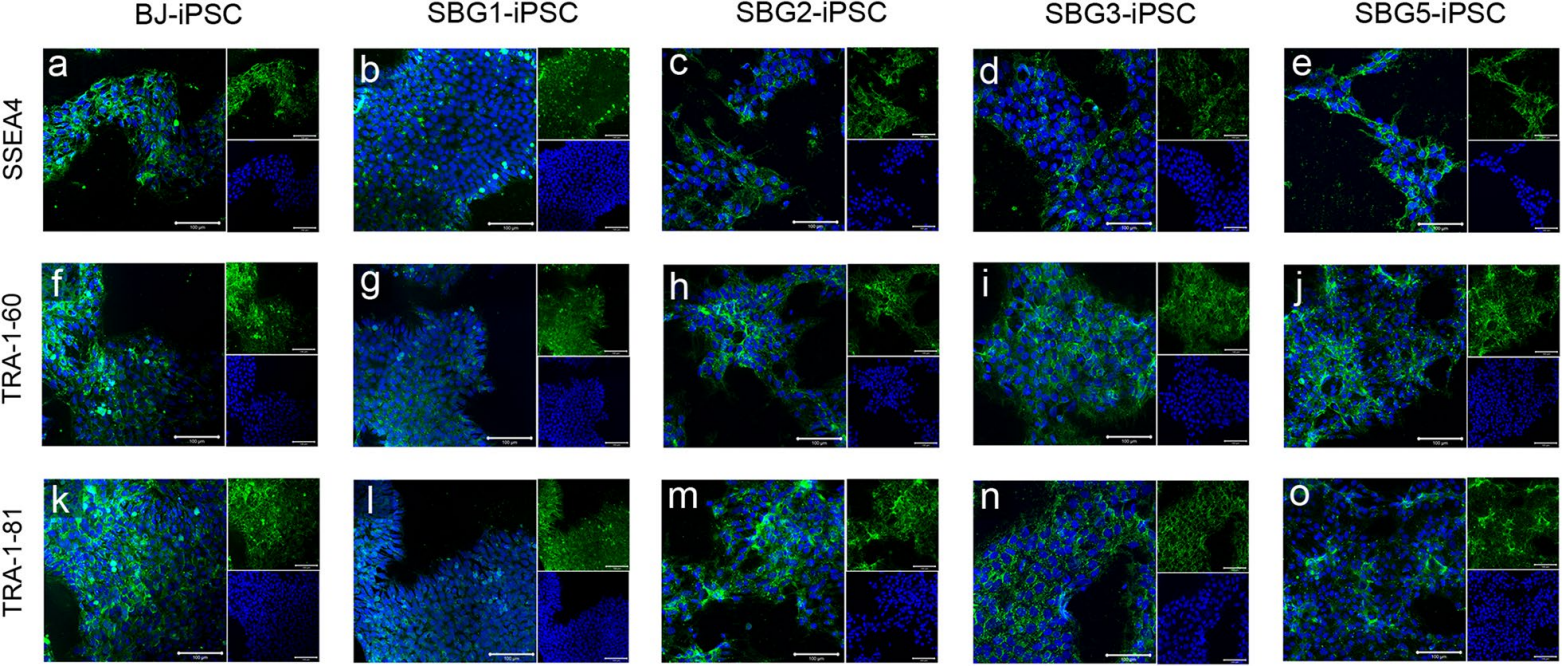
Mitochondrial disease patient hiPSCs express cell surface pluripotency markers. SSEA4, TRA-1-60 and TRA-1-81. Healthy control BJ-hiPSC (**a**, **f**, **k**) and diseased SBG1-hiPSC (**b**, **g**, **l**), SBG2-hiPSC (**c**, **h**, **m**), SBG3-hiPSC (**d**, **i**, **n**) and SBG5-hiPSC **e**, **j**, **o** are positive for SSEA4 (**a**–**e**), TRA1-60 **f**–**j** and TRA-1–81 (k–o) cell surface markers. Image inserts contain corresponding DAPI nuclear counterstain. DAPI, 4’,6-diamidino-2-phenylindole. Scale bar = 100 µm

Phase contrast images revealed putative patient-derived SBG1-3,5-hiPSC lines displayed typical hiPSC morphology including high nucleus to cytoplasm ratio and large, prominent nucleoli comparable to healthy control BJ-hiPSC line (Fig. 2). Putative hiPSCs were assessed for the expression of pluripotency markers. Immunocytochemistry analysis of patient-derived hiPSCs with antibodies directed against POU5F1 (aka OCT4) and SOX2 demonstrated that these pluripotency transcription factor proteins were robustly expressed and correctly localized to the nucleus in a comparable manner to CTL-BJ-hiPSCs (Fig. 2). Flow cytometry analysis showed POU5F1, SOX2 and NANOG were a co-expression > 95% of analyzed control BJ-hiPSCs, and > 97% of analyzed hiPSC1-3, 5 cells (Additional file 1: Figure S1). Patient-derived hiPSC1-3, 5 cells were also assessed for the canonical hiPSC cell surface glycoprotein and glycolipid epitopes stage-specific embryonic antigen (SSEA)-4, tumor-related antigen (TRA)-1-60 and TRA-1-81 [41, 59, 60]. Immunocytochemistry showed robust expression of SSEA-4, TRA-1-60 and TRA-1-81 in hiPSC1-3, 5 and at comparable levels to CTL-BJ—hiPSCs (Fig. 3). Flow cytometry analysis showed patient-derived hiPSCs expressed pluripotency surface markers SSEA4, TRA-1-60 and TRA-1-81 (Additional file 1: Figure S2). SSEA-4 was expressed in 98.2% of SBG1-hiPSC, 93.6% of SBG2-hiPSC, 95.6% of SBG3-hiPSC, 97.2% of SBG5-hiPSC and 98.5% of CTL—BJ-hiPSCs. TRA-1-60 was expressed in 98.7% of SBG1-hiPSC, 98.6% of SBG2-hiPSC, 99.1% of SBG3-hiPSC, 98.4% of SBG5-hiPSC and 96.8% of BJ-hiPSCs. TRA-1-81 was expressed in 88.8% of SBG1-hiPSC, 82.7% of SBG2-hiPSC, 94.5% of SBG3-hiPSC, 87.1% of SBG5-hiPSC and 83.1% of CTL-BJ-hiPSCs. Taken together, immunocytochemistry and flow cytometry data show activation of the endogenous pluripotency network and reprogramming of mitochondrial disease patients’ fibroblasts into hiPSCs.

### Karyotype analysis demonstrated no aneuploidies or significant structural abnormalities

An important feature of creating hiPSCs is the ability to generate cell models with normal karyotypes indicative of stable nuclear genome [61]; so that further studies can be conducted to measure the effect of mitochondrial genome perturbations on mitochondrial function without chromosomal abnormalities confounding data interpretation or causing lethal phenotypes. In our study, karyotypes were created three times with a total of at least 20 metaphases counted and imaged for each cell line to determine if reprogramming led to karyotypic abnormalities. At the band level analyzed (400–450), aneuploidies and significant structural chromosome abnormalities present in the cell lines can be evaluated. Our analysis indicated that no aneuploidies or significant structural abnormalities were present in the hiPSC lines (Additional file 1: Figure S3).

### Sanger sequencing demonstrates presence of mutations in hiPSCs

To confirm the presence of the mutation in all the generated hiPSCs, the mtDNA was extracted from whole cell pellets, and specific regions of interest within the ATP6 gene (for SBG1-hiPSC and SBG2-hiPSC: 8993 T > G, SBG3-hiPSC: 9185 T > C) and ND5 gene (for SBG5-hiPSC: 12706 T > C) were PCR amplified using primers detailed in (Additional file 1: Table S1).

**Fig. 4:**
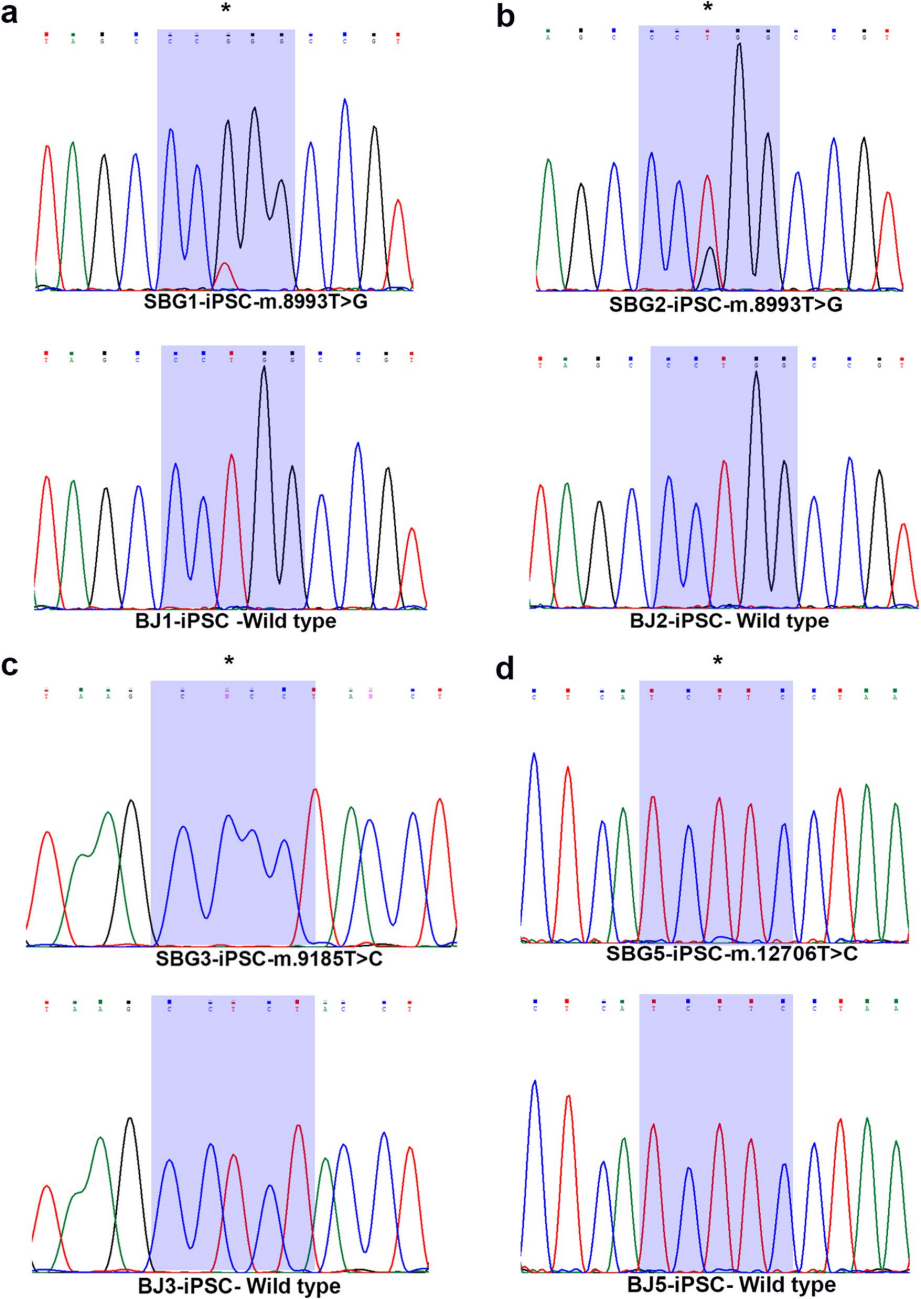
Detection of mutation through PCR amplification and Sanger sequencing of control, healthy BJ-hiPSC and diseased SBG1, 2, 3, 5-iPSC. Following extraction of mtDNA, detection of mutation through PCR amplification and Sanger sequencing of mtDNA was conducted in **a** SBG1-hiPSC (8993 T > G) **b** SBG2-hiPSC (8993 T > G) **c** SBG3-hiPSC (9185T > C) and (**d**) SBG5-hiPSC (12706 T > C). mtDNA, mitochondrial DNA. Mutation was detected in all diseased hiPSCs while absent in the control, healthy BJ-hiPSCs

After the PCR products were purified and quantified to the appropriate specification, Sanger sequencing was necessary to confirm the presence of the disease-causing mutation after reprogramming. The results demonstrate that the 8993 T > G mutation is present in SBG1-hiPSC and SBG2-hiPSC lines (Fig. 4a, b); the 9185 T > C mutation is present in the SBG3-hiPSC line (Fig. 4c); and the 12706 T > C mutation is present in the SBG5-hiPSC line (Fig. 4d). The control BJ-hiPSC samples were found to be devoid of these specific point mutations.

### Next-generation sequencing demonstrates presence of variable heteroplasmy burden in hiPSCs

With the presence of the mutation confirmed in all hiPSCs by Sanger sequencing, the percentage of mutation burden in each sample was measured using high-throughput next-generation sequencing for whole exome. This strategy has previously been utilized to effectively measure heteroplasmies in the mitochondrial genome [62–64] and in our previous study focused on generation of hiPSCs using non-viral reprogramming approaches [32]. In our current study, the mtDNA from different samples were extracted from whole cell pellets and then purified before sequencing. The sequencing results yielded a range of total reads to be analyzed between 213 and 1399 in the different cell samples. This large sample size allows us to be confident about the percentages measured. Our results indicate that SBG3-hiPSC exhibited a high level of the mutation burden (96%), with SBG1-hiPSC and SBG2-hiPSC exhibiting 82% and 24% respectively. Although the Sanger sequencing confirmed the presence of the mutation in SBG5-hiPSC, the mutation burden based on next-generation sequencing was extremely low (1%) (Table 2).

**Table 2:** Quantification of heteroplasmy by next-generation sequencing

Sample name	Mutation	Total number analyzed	Normal	Variant	Percentage of mutation (%)
SBG1-hiPSC	8993 T > G	213	38 (T)	175 (G)	82
SBG2-hiPSC	8993 T > G	1399	1058 (T)	341 (G)	24
SBG3-hiPSC	9185 T > C	548	23 (T)	525 (C)	96
SBG5-hiPSC	12706 T > C	493	489 (T)	4 (C)	1

The extracted mtDNA was next-generation-sequenced for whole exome. The sequencing results were compiled, and the results were analyzed as detailed in the methods section. This allows us to compute heteroplasmic variants based on the sequencing reads. Results demonstrate the presence of pathogenic mtDNA burden in SBG1-3 hiPSC, while very low in SBG5-hiPSC

### Metabolic comparison of oxidative phosphorylation of BJ-hiPSCs and SBG1-3, 5-hiPSCs

**Fig. 5:**
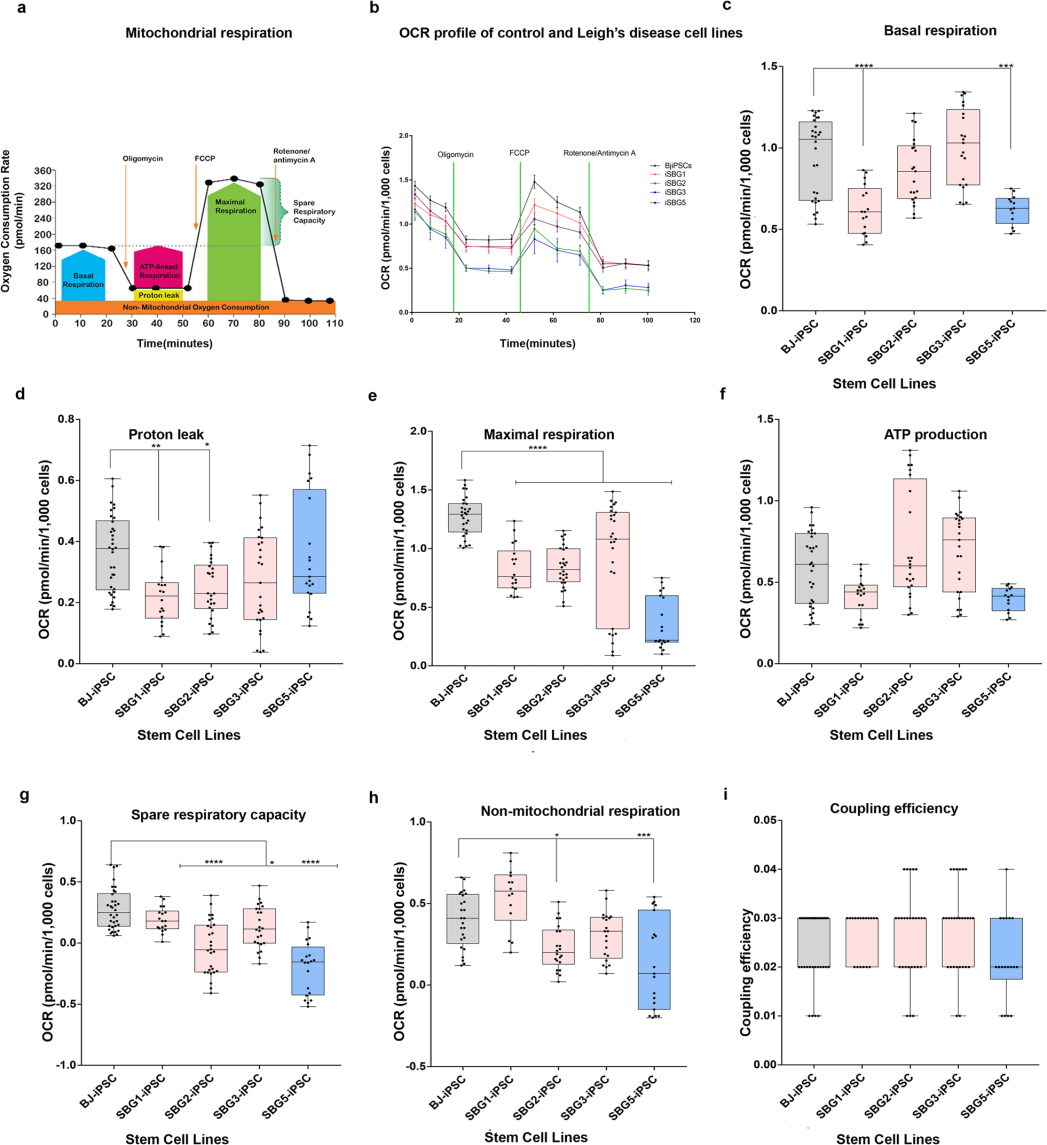
Mitochondrial respiratory profile of hiPSC cell lines. **a** Scheme of expected oxygen consumption rate (OCR) under basal conditions, **b** OCR profile of BJ-iPSC and diseased (SBG1, 2, 3, 5) hiPSCs, **c** basal respiration, **d** proton leak, **e** maximal respiration, **f** mito-ATP production after oligomycin injection **g** spare respiratory capacity after FCCP injection, **h** non-mitochondrial respiration after Rot/AA injection, **i** coupling efficiency. All parameters are in pmol/min/1000 cells. Data are presented as mean ± SD. Experiments were repeated at least three times on different days under the same conditions. *p < 0.05**p < 0.01***p < 0.001****p < 0.00001. Comparative analyses for all diseased (SBG1, 2, 3, 5) hiPSCs were conducted with the healthy control BJ-hiPSC line

Tracing of cell respiratory control is the most useful technique to reflect mitochondrial function in a cellular population and to highlight any mitochondrial dysfunction. In a single experimental run, several mitochondrial functional parameters are measured by the addition of mitochondrial inhibitors and uncouplers. The generic mitochondrial respiration profile via measurement of oxygen consumption rate (OCR) in a normal functioning cell population that is subject to the mito-stress test is detailed in Fig. 5a. Briefly, the purpose of the test is to measure respiration under basal conditions and subsequently expose the cells to several stressors, such as sequential addition of oligomycin (ATP synthase inhibitor) that allows measurement of OCR that is the result of non-mitochondrial respiration and proton leak; FCCP (uncoupler) that permits measurement of the maximum state of mitochondrial respiration via the ETC independent of ADP phosphorylation; and finally rotenone and antimycin A (complex I and III inhibitors respectively) which completely block the respiratory ETC leading to the ability to measure non-mitochondrial background and thus calculate mitochondrial respiration. Results indicate that similar to previously published studies [52, 65, 66], the control healthy BJ-hiPSCs demonstrate a lower response to oligomycin and FCCP when compared with the parental BJ fibroblast (data not shown) which may indicate decrease in spare capacity of these cells and increase in the non-mitochondrial respiration with a relatively high proton leak, upon reprogramming of the BJ fibroblasts to BJ-hiPSCs.

The representative time course data for all the cell lines (*n* = 3–5) are shown in Fig. 5b. Based on OXPHOS analysis, the diseased SBG1-3,5 hiPSCs demonstrated lower mitochondrial dependency than the control healthy BJ-hiPSC line. Results indicate that the diseased hiPSCs exhibited reduced basal respiration when compared with BJ-hiPSCs, with statistically significant reduced respiration in SBG1-hiPSC (34% decrease; *p* = 0.00001) and SBG5-hiPSC (33% decrease; *p* = 0.0001) (Fig. 5c). All diseased hiPSCs also exhibited reduced proton leak when compared with BJ-hiPSCs, with statistically significant reduced proton leak in SBG1-hiPSC (39% decrease; *p* = 0.0035) and SBG2-hiPSC (31% decrease; *p* = 0.0119) (Fig. 5d). However, the maximal respiration was significantly reduced in all diseased lines (SBG1-34% decrease; *p* = 0.00001; SBG2-33% decrease; *p* = 0.00001; SBG3-26% decrease; *p* = 0.00001; SBG5-71% decrease; *p* = 0.00001) when compared with BJ-hiPSCs (Fig. 5e). Given that ATP is primarily produced in hiPSCs by glycolysis, the mitochondrial ATP production was not significantly different between the control BJ-hiPSCs and all diseased hiPSCs despite the reduced functionality of the ATP synthase (Fig. 5f). In line with reduced maximal respiration, the spare respiratory capacity was significantly decreased in all the diseased hiPSCs and non-significantly in SBG1-hiPSC (SBG2-113% decrease; *p* = 0.00001; SBG3-52% decrease; *p* = 0.023; SBG5-172% decrease; *p* = 0.00001) when compared with the control BJ-hiPSC (Fig. 5g). The non-mitochondrial respiration was significantly reduced in SBG2-hiPSC (42% decrease; *p* = 0.0245) and SBG5-hiPSC (67% decrease; *p* = 0.0001), reduced in SBG3-hiPSC, while slightly higher in SBG1-hiPSC when compared with BJ-hiPSC (Fig. 5h). No significant change was noticed in coupling efficiency in all diseased hiPSCs **(**Fig. 5i).

### Metabolic comparison of glycolytic profile of BJ-hiPSCs and SBG1 to 3, 5-hiPSCs

**Fig. 6:**
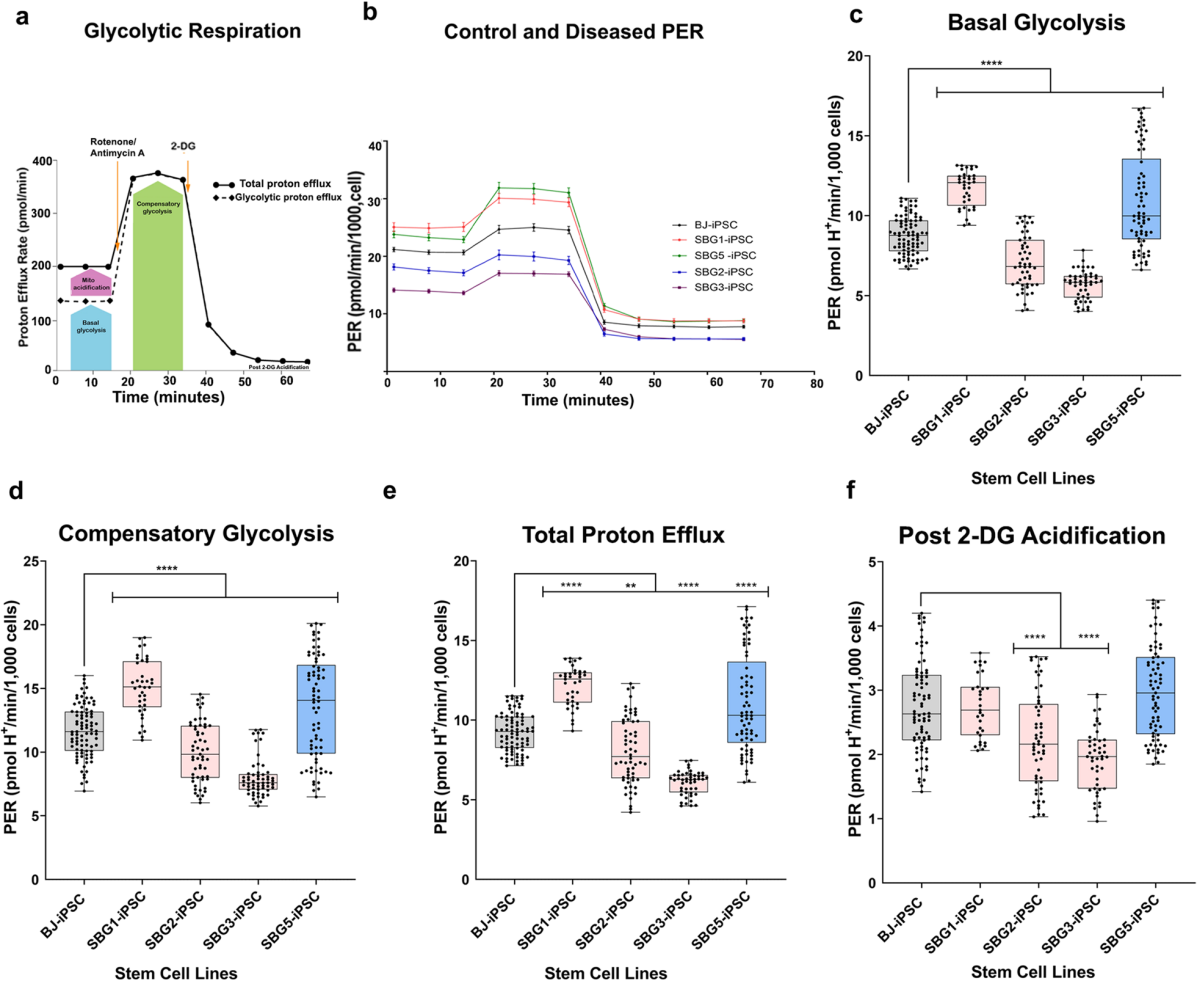
Glycolytic profile of hiPSC lines. **a** a scheme shows the glycolytic acidification, **b** PER of BJ-iPSC and diseased (SBG1, 2, 3, 5) hiPSCs, **c** basal glycolysis, **d** compensatory glycolysis after ETC blocking using Rot/AA, **e** total proton efflux rate, **f** post-2-DG (non-glycolytic acidification). Data are shown in pmol H + /min/1000 cells as mean ± SD. Experiments were repeated at least three times on different days under the same conditions. *p < 0.05**p < 0.01***p < 0.001****p < 0.00001. Comparative analyses for all diseased (SBG1, 2, 3, 5) hiPSCs were conducted with the healthy control BJ-hiPSC line. PER: proton efflux rate

The preference of glycolysis over OXPHOS under both normoxic and hypoxic conditions is a key characteristic of hiPSCs [52]. Glycolysis measurements using the extracellular flux analyzer provide readouts of proton efflux rate (PER). Concurrent addition of the ETC inhibitors rotenone and antimycin A permits PER measurements in basal glycolysis, while inhibition of glycolysis by 2-deoxy-glucose (2-DG) allows measurement of other parameters like compensatory glycolysis, total proton efflux and post-2-DG acidification (Fig. 6a). The representative time course data for all the cell lines (*n* = 3–5) are shown in Fig. 6b. Overall, the PER profile results indicate a significant difference in the glycolytic profile in the diseased hiPSCs when compared with the control healthy BJ-hiPSCs, with a different compensatory mechanism for each cell line indicating the possibility that the reprogrammed hiPSCs are potentially affected by the presence of the mutation and also by their original physiological environment and epigenetic memory of the respective parental patient fibroblasts (Fig. 6b). Both SBG1-hiPSC (33% increase; *p* = 0.00001) and SBG5-hiPSC (24% increase; *p* = 0.00001) exhibited a statistically significant increase in basal glycolysis under basal conditions and a statistically significant increase in compensatory glycolysis (SBG1-29% increase; *p* = 0.00001; SBG5-15% increase; *p* = 0.00001), after inhibition of ETC by (Rot/AA) (Fig. 6c, d), when compared with control BJ-hiPSCs. The observed increase in basal and compensatory glycolysis in SBG1-hiPSC and SBG5-hiPSC is potentially a compensation mechanism in response to the reduced mitochondrial oxygen consumption and mitochondrial ATP production (Fig. 6c and e). However, both SBG2-hiPSC (19% decrease; *p* = 0.00001) and SBG3-hiPSC (35% decrease; *p* = 0.00001) exhibited a statistically significant decrease in basal glycolysis under basal conditions and a statistically significant decrease in compensatory glycolysis (SBG2-14% decrease; *p* = 0.001; SBG3-31% decrease, *p* = 0.00001) after inhibition of ETC by (Rot/AA) (Fig. 6c, d), when compared with control BJ-hiPSCs. Results thus indicate that both SBG1-hiPSC and SBG5-hiPSC are highly dependent on glycolysis and less on OXPHOS pathways, while SBG2-hiPSC and SBG3-hiPSC are dependent on both OXPHOS and glycolysis pathways. The total proton efflux which include both glycolytic and mitochondrial acidification follow the same trend (SBG1-31% increase, *p* = 0.00001; SBG2-12% decrease, *p* = 0.0015; SBG3-34% decrease, *p* = 0.00001; SBG5-19% increase, *p* = 0.00001) as basal and compensatory glycolysis with all the diseased cell lines (Fig. 6e); reflecting the limited share of mitochondrial acidification into the total PER. Non-glycolytic acidification after inhibition of glycolysis using 2-DG showed no statistically significant increase in SBG1-hiPSC and SBG5-hiPSC, while demonstrating a statistically significant decrease in SBG2-hiPSC (18% decrease, *p* = 0.00001, SBG3-hiPSC (29% decrease, *p* = 0.00001) when compared with control BJ-hiPSC (Fig. 6f).

### Differentiation outcomes of SBG1 to 3, 5-hiPSCs

**Fig. 7:**
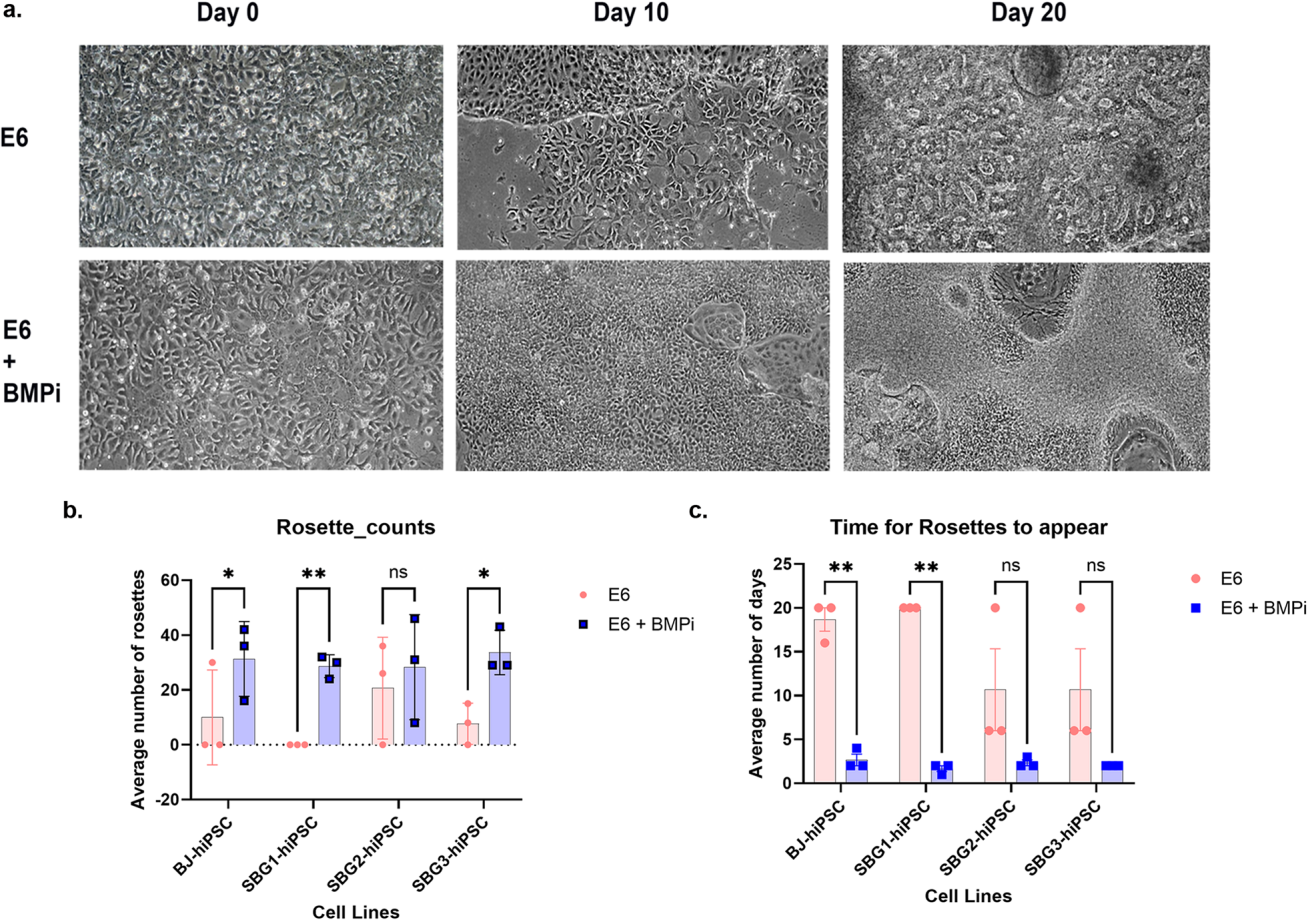
LS-hiPSC cells differentiation into neurons. **a** Representative phase contrast images for SBG2-hiPSCs on day 0 of differentiation using medium 1-E6 and medium 2-E6 + BMPi. On day 10 of differentiation using Medium 2-E6 + BMPi, rosettes were generated. Day 20 of differentiation using medium 1-E6, SBG2-hiPSCs had formed a larger colony of neural rosettes. While using medium 2-E6 + BMP, SBG2-hiPSCs exhibited neural projections from the larger cell colonies. **b** A bar graph showing that Medium 2 (E6 + BMPi) led to a significant increase in the average number of rosettes for SBG1 and SBG3 compared to Medium 1-E6 after 20 days. **c** Bar graph of the average time for neural rosettes to appear for Medium 1 (E6) was greater than Medium 2 (E6 + BMPi). *p < 0.05**p < 0.01***p < 0.001****p < 0.00001

**Fig. 8:**
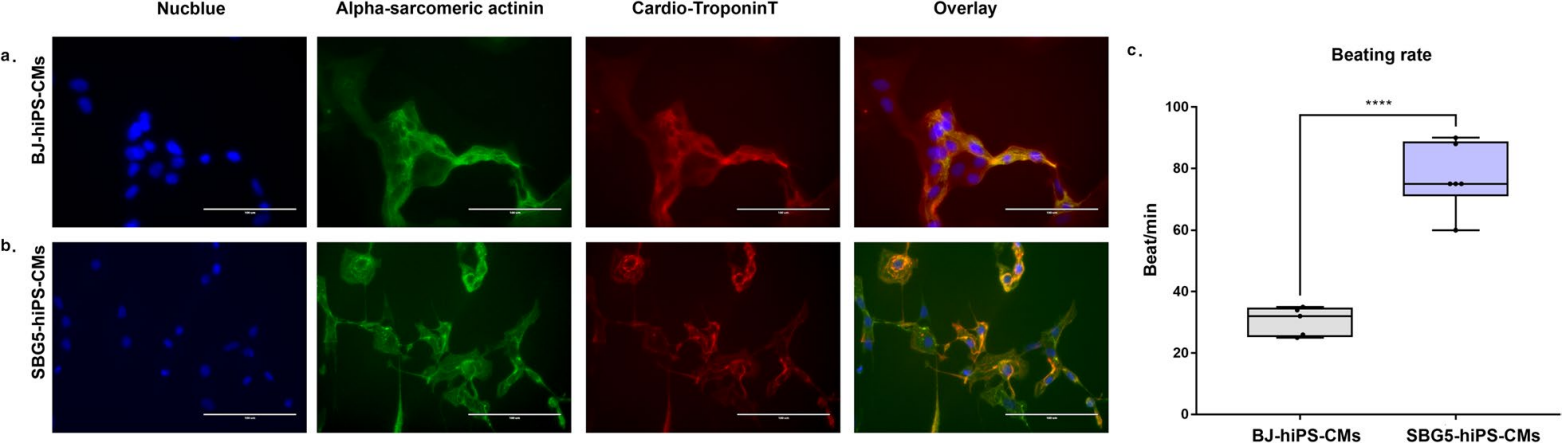
SBG5-hiPSCs differentiation into cardiomyocytes (CMs). Representative fluorescent images of positive immunocytochemistry stain of cardiac markers such as α-sarcomeric actinin (green) and cardiotroponin T (red) **a** BJ-hiPSC-CMs and (**b**) SBG5-hiPSC-CMs. Nucleus was counterstained with Nucblue (blue), and overlaid images were created. **c** A graph figure of beating frequency calculated by counting beats per minute in SBG5-hiPS-CMs in comparison to control BJ-hiPSC-CMs. ****p < 0.0001. Scale bar = 100 µm

Cells isolated from patients with ATP synthase mutation such as SBG1-3 were presented with symptoms denoting neuronal involvement such as LS, abnormal gait and myopathy. Thus, in the context of this study, SBG1-3 hiPSCs were differentiated into neurons. We used a standard neural rosette differentiation protocol that has been frequently published [53, 67, 68] and are confident that the differentiation outcomes lead to neural rosette formation, based on their characteristic morphology that is not exhibited by other non-neural differentiation outcomes. The focus of this manuscript was to conduct analysis of preliminary differentiation outcomes. As an example, we show the phase contrast representative images for SBG2-hiPSCs, during the differentiation process. We specifically assessed the potential for differentiation using two different medium formulations (Medium 1-E6; Medium 2-E6 + BMPi). Our results indicate that after five days of differentiation, Medium 1-E6 did not produce any neural rosettes, while neural rosettes were minimally observed using Medium 2-E6 + BMPi (data not shown). In addition, after ten days of differentiation using Medium 2-E6 + BMPi, rosettes were generated (Fig. 7a) and were abundant by Day 15 (data not shown). However, after 20 days of differentiation using Medium 1-E6, SBG2-hiPSCs had formed a larger colony of neural rosettes (Fig. 7a), while use of Medium 2-E6 + BMPi led to SBG2-hiPSCs exhibiting neural projections from the larger cell colonies (Fig. 7a). Overall, application of medium 2 (E6 + BMPi) led to a significant increase in the average number of rosettes for SBG1 and SBG3 compared to Medium 1-E6 after 20 days (Fig. 7b). In general, the average time for neural rosettes to appear for Medium 1 (E6) was greater than Medium 2 (E6 + BMPi) **(**Fig. 7c**),** thus indicating that addition of BMPi was necessary and endorsed neural differentiation in diseased hiPSCs. Patients harboring mitochondrial mutation in complex I of the electron transport chain are usually presented with cardiac manifestation [26, 69]. Thus, we sought to differentiate SBG5-hiPSCs into cardiomyocytes (CM). Immunocytochemical analysis demonstrated cardiac marker expression such as cardio-troponin T and α-sarcomeric actinin (Fig. 8a) in both control BJ-hiPSC-CMs (Fig. 8a) and SBG5-hiPSC-CMs (Fig. 8b). At day 30, unpurified population of SBG5-hiPS-CMs shows significantly faster beating frequency than control BJ-hiPSC-CMs (*p* < 0.0001) **(**Fig. 8c**),** indicative of aberrant differentiation outcomes.

## Discussion

The hiPSC lines in this study were generated from patients’ fibroblasts suffering from LS mitochondrial diseases [28, 29]. The obtained cells exhibited many classic hallmarks of a pluripotent stem cell, including typical hiPSCs morphology such colony formation and high nucleocytoplasmic ratio in addition to the high expression of pluripotency-associated markers like POU5F1, SOX2, NANOG, SSEA4, TRA1-60 and TRA1-81. Moreover, all the hiPSCs were generated using a non-viral reprogramming approach with a cocktail of non-modified reprogramming and immune evasion mRNAs and microRNAs and exhibited a normal karyotype. Our results are in concordance with our earlier study which demonstrated the derivation and characterization of a stable non-viral hiPSC line reprogrammed from a LS patient fibroblast [32]. In addition, the use of non-viral and non-integrating xeno-free mRNA and miRNA reprogramming approach enhances the preclinical significance of our hiPSCs, as the clinical grade hiPSCs could be used to derive specific differentiated cell types for potential use in cell replacement and regenerative biomedical therapies.

Our methods included the use of Sanger sequencing and next-generation sequencing to assess the presence of the mtDNA mutation and to also quantify the mutation burden. Sanger sequencing confirmed the presence of the mutation in all reprogrammed hiPSCs, while next-generation sequencing demonstrated variability in the mutation burden in the generated hiPSCs. This finding is in line with previous studies that have demonstrated the potential for reprogramming to generate hiPSCs with varying degrees of heteroplasmy [39]. However, generating hiPSCs with the disease-specific mutations is an important step in further confirming non-viral, non-integrating reprogramming technologies as a viable option of creating karyotypically stable cell-based models for mitochondrial disorders.

Given the clinical variability among patients with mitochondrial disorders, the generation and characterization of patient-specific hiPSCs represent an important first step in producing a stable source of cells for further differentiation into specialized cell types like neurons and cardiomyocytes that are frequently affected in LS patients. Given the clinical complexity in mitochondrial diseases, we hypothesized that hiPSCs harboring mtDNA mutation in genes affecting complex I and V would exhibit mitochondrial dysfunction and correlate with clinical pathophysiology in neuronal and cardiac lineage seen in LS patients. Valuable insights into the bioenergetics of the hiPSCs was obtained by measuring the OCR and PER using the XFe96 extracellular flux analyzer. We were able to measure the OCR as an indicator for mitochondrial respiration and PER for glycolysis. By using different mitochondrial inhibitors, the bioenergetic profile of the generated hiPSCs was analyzed for different parameters including basal respiration, ATP turnover, maximal respiration and spare respiratory capacity.

Overall, our results demonstrate that hiPSCs (both control BJ and diseased SBG1,2,3,5) have a greater reliance on glycolysis (Fig. 6c) than mitochondrial respiration (Fig. 5c), which is in line with energy metabolism exhibited by hPSCs [66]. In accordance with previously published data, our results indicate that BJ-hiPSCs exhibit similar basal OCR and spare reserve capacity with other hESC and hiPSC lines [66]. More importantly, we demonstrate that even though healthy and diseased hiPSCs rely on glycolysis, the different hiPSCs generated from patient fibroblasts are not identical in terms of oxygen consumption and basal glycolysis rates. Specifically, SBG1,5-hiPSCs exhibited similar bioenergetic profiles with low mitochondrial respiration and high glycolysis when compared with healthy control BJ-hiPSCs. However, SBG2,3-hiPSCs exhibited low mitochondrial respiration and low glycolysis when compared with healthy control BJ-hiPSCs.

In accordance with previously published data, our results indicate that BJ-hiPSCs exhibit similar basal OCR and spare reserve capacity with other hESC and hiPSC lines [66]. To further assess the implication of glycolysis and OXPHOS in the diseased hiPSCs, we computed the ratio of ATP derived by mitochondrial oxygen consumption over ATP derived by glycolysis (Additional file 1: Figure S4). We also took into consideration the significance of the ‘spare reserve capacity’ as it is a good indicator of the amount of extra ATP that can be produced by OXPHOS in case of a sudden increase in energy demand in response to stress or increased workload. Depletion of reserve capacity has been implicated in a range of pathologies affecting high-energy tissues (e.g., brain, heart and skeletal muscle) because these cells are unable to provide the required ATP and risk being driven to death [70].

Based on analysis of the four generated diseased hiPSCs, elevated glycolysis contributed to an increased rate of proliferation in hiPSCs, while disease severity influenced mitochondrial ATP levels and factors regulating spare reserve capacity. For example, in the SBG5-hiPSC (*MT-ND5*-12706 T > C) cell line, the maximal respiration (Fig. 5e) and spare respiratory capacity (Fig. 5g) were significantly reduced when compared to the healthy control BJ-hiPSC. In order to compensate for the loss in mitochondrial ATP production by OXPHOS, we anticipate that the patient-specific SBG5-hiPSCs tried to increase glucose uptake in a futile attempt to increase glycolysis derived ATP (Fig. 6c). In SBG1-hiPSC (*MTATP6*-8993 T > G), the maximal respiration (Fig. 5e) was significantly reduced by 34% when compared to the healthy control BJ-hiPSC. However, the spare respiratory capacity was not significantly different from the healthy control BJ-hiPSC, which indicated that the cells could overcome a sudden energy demand. Since the mitochondrial ATP content in SBG1-hiPSC were low (Fig. 5f) when compared with BJ-hiPSC, the patient cells would compensate for this shortage by increasing compensatory glycolysis (Fig. 6d) and total proton efflux rate measurement (Fig. 6e). However, in SBG2-hiPSC (*MTATP6-*8993 T > G) which has the same mutation as SBG1, cell respiration assays confirmed that both the spare reserve capacity (113%) and maximal respiration (33%) was significantly lower than the healthy BJ-hiPSC (Fig. 5g). In addition, the mitochondrial ATP was slightly elevated (Fig. 5f), which did not appear to trigger the cells to increase basal glycolysis (Fig. 6c) or total proton efflux (Fig. 6e). Finally, in SBG3-hiPSC (*MT-ATP6*-9185 T > C), the spare reserve capacity (52%) and maximal respiration (26%) were significantly lower in the SBG3-hiPSC than the healthy BJ-hiPSC (Fig. 5g), the mitochondrial ATP was slightly elevated (Fig. 5f) and thus did not require the cells to increase glucose uptake as an adaptive mechanism for survival, and potentially avoiding more lactic acid buildup. This observation may also be to a certain extent related to the age at which the disease was presented. A study in a drosophila model of ATP6 dysfunction shows a dramatic increase in lactic acid and glycolytic rate at early age of the disease onset, while later in life, these glycolytic levels declined to the wild-type levels [71]. Given that SBG3-hiPSCs were obtained from a 23-year-old patient [28], our results are in line with this previous study that an increased glycolysis is an important early compensatory mechanism. However, this increase is ineffective at fully terminating the reduced bioenergetics prior to disease pathogenesis. These results also suggest that lactic acidosis is not likely to account for the severe pathogenesis observed late in life particularly in ATP synthase dysfunction [71].

Results from our previous studies on the parental fibroblast lines showed that the disease severity was associated with specific bioenergetic parameters [28]. Specifically, the spare reserve capacity (SRC) and the glycolytic rates observed in the generated hiPSCs (Additional file 1: Figures S4 and S6) align with the bioenergetic health index ratio observed in the parental fibroblasts [28]. SRC represents the mitochondrial capacity to meet additional energy requirements, beyond the basal level in response to different cellular stress events and thereby preventing an ATP crisis [28, 66, 72, 73]. In our previous studies on the parental fibroblasts, patient cells with low SRC had the severe clinical presentation and the lowest survival rate [28]. Our current study notes that two of the cell lines (SBG5-hiPSCs and SBG2-hiPSCs) exhibited significant reduction in SRC (SBG2-hiPSCs-113% decrease; *p* = 0.00001; SBG5-hiPSC 172% decrease; *p* = 0.00001) when compared with the control BJ-hiPSC (Additional file 1: Figures S4 and S5g). Specifically, in the parental fibroblast, we observed a statistically significant reduction in SRC in SBG2-FBs (11%, *p* < 0.0001) and in SBG5-FBs (50%, *p* < 0.0001), when compared with the healthy control BJ-FBs [28]. Taken together, this data is likely to characterize the clinical presentation (Table 1) at the earliest stages of development as represented by the hiPSCs which is considered as ‘ES-like’ and in vitro equivalents of the developing embryo. More importantly, the decreased SRC values in SBG2 & SBG5-hiPSCs are in alignment with the decreased SRC values observed in the parental ‘differentiated’ SBG2 & SBG5-FB fibroblast cells [28]. Given that mitochondrial function is a highly dynamic process and SRC represents the mitochondrial capacity to meet extra-energy requirements, beyond the basal level; our findings correlating the SRC with clinical presentation and survival in the generated hiPSC lines are highly significant in the development of relevant in vitro models to better understand the origins of LS and understanding mitochondrial dysfunction in early development stages of LS patients.

A common view of mitochondrial diseases harboring mtDNA variants is there is a direct correlation between clinical symptoms and outcomes to the degree of heteroplasmy. Many studies have alluded to a high degree of heteroplasmy (> 90%) leading to LS, whereas a lower heteroplasmy (70–90%) results in a neuropathy, ataxia and retinitis pigmentosa (NARP) phenotype [74]. However, our results based on comprehensive bioenergetic analysis and disease severity did not strongly correlate with the observed heteroplasmy levels in the mtDNA mutations (*MT-ATP6*: 8993 T > G; *MT-ATP6*: 9185 T > C; *MT-ND5*: 12706 T > C) (Table 1 and Additional file 1: Table S2). Similarly, our results previously indicated varying heteroplasmy levels (between 39 and 98%) in the different fibroblasts [28]. Our findings are in line with a recent study that involved the large group of patients with mitochondrial *MT-ATP6* mutations, where a detailed analysis of the clinical and genetic information from 132 mutation carriers from national registries and local databases from Europe, USA, Japan and China was directed [75]. In this study, the authors noted that the phenotypic spectrum ranged from asymptomatic to early onset multisystemic neurodegeneration and the degree of mutation heteroplasmy did not reliably predict disease severity. Another recent study that focused on interpreting mtDNA variants indicated that key biological factors that uniquely complicate mtDNA variant interpretation include mtDNA haplogroups and heteroplasmy, with variable spatio-temporal changes that could be subject to variant-specific or tissue-specific thresholds and underlie a broad spectrum of phenotypes that may differ between individuals [76].

After reprogramming the fibroblasts isolated from LS fibroblasts, we proceeded with neuronal differentiation of ATP synthase defects hiPSCs and cardiogenic differentiation of complex I defect hiPSCs to test their directed differentiation potential and any associated dysfunction that can observed due to the mutations and may correlate with the clinical disease pathophysiology. Complex I mutations are usually associated with cardiac involvement and impairment in cardiac function [26, 77, 78]. A recent study on cells with complex I mutation (*m.13513G* > *A*) showed that cardiac differentiation is impaired in these cells [77]. In our study, while SBG5 (m.*12706 T* > *C)* hiPSCs were directly differentiated into cardiomyocytes, they show a higher abnormal beat when compared to BJ-hiPSC-CMs. Also, hiPSCs harboring ATP synthase mutation showed difficulty in differentiating into neurons in the absence of specific growth factors. These observations may highlight the preference of different mitochondrial mutations to organs altered and into the clinical features presented in each patient (Table 1).

Thus, our study for the first time clearly demonstrates the need for comprehensive bioenergetic analyses in mitochondrial diseased patient-derived hiPSCs and subsequently in differentiated tissue-specific disease models as a means to better understand the complexities associated with early developmental and mitochondrial disorders. In addition, comparative bioenergetics analysis has been conducted with one healthy control fibroblast (BJ-hiPSC) cell line, derived from BJ-FB. The choice of BJ-FB cell line was based on its neonatal origin. In order to keep our experimental design simple and consistent across multiple projects and to minimize variability, we decided to use only one control cell line across all our methodologies for comparing with disease patient-derived iPSCs related to ‘pediatric’ mitochondrial disorders. Future studies will enable a better understanding of the role of pathogenic mtDNA mutations and their variants in high-energy-consuming tissues such as the brain, heart and muscle that have not undergone rigorous systematic evaluation. Specifically, hiPSCs will be differentiated into neural, cardiovascular and muscle cells, commonly affected in LS. Comparison of bioenergetics status and heteroplasmy of the differentiated derivatives with parental fibroblasts will allow for a better understanding of changes that occur during cell fate and differentiation. In addition, these LS-specific differentiated cells will be evaluated for changes in development (e.g., neurite outgrowth) and functional phenotypes (e.g., electrical activity) and correlated with bioenergetics and mutant heteroplasmy. Thus, use of patient-derived hiPSCs and controlled directed differentiation into specialized cell and tissue types will contribute to a better understanding of genotype, phenotype and clinical variability associated with mitochondrial disorders.

Despite the significant advancements highlighted in this study, there are some limitations. In spite of hiPSCs serving as relevant in vitro models for better understanding early development and differentiation, it is important to recognize that hiPSCs can exhibit variability in culture that could be attributed to genetic and epigenetic changes during the reprogramming process [79–88]. Many strategies to reduce variation to overcome the genomic mutation risk caused by viral integrative methods have been proposed that include use of non-viral mRNA and miRNA—based reprogramming approaches such as what has been used in this study [89]. It is important to recognize that this study used only one healthy control, and thus, our findings are limited as variability within healthy controls cannot be excluded. In our future studies, adoption of isogenic approaches to better examine the effect of variants with specific genetic backgrounds and use of single-cell assay technologies is expected to address this limitation [79]. In this study, we also performed an initial evaluation of how mtDNA mutations that are present in mitochondrial disorders affect neural and cardiomyocyte differentiation outcomes. Neural differentiation outcomes were exclusively based on rosette formation and no neuronal markers were utilized. In future studies, we plan to further examine how specific mitochondrial defects in mtDNA genome that include point mutations and deletions effect key morphological features such as neurite outgrowth and synapse formation in diseased neuronal cells and functional characteristics like action potentials, firing rates and synchronization in derived neurons and cardiomyocytes. In addition, it is important to recognize that even though mitochondrial disorders primarily impact children, there are instances where the clinical manifestations show up later in life. It is thus important for future studies to evaluate gender and age as variables and how differentiation outcomes could be altered as result. It is our expectation that our follow-up studies will provide critical insights into how changes in cellular form and function lead to disease pathology and provide us an opportunity to screen and evaluate novel therapeutics that may limit these detrimental changes.

## Conclusions

In summary, we established multiple hiPSCs from patients exhibiting early developmental and mitochondrial disorders. The study has identified the need for comprehensive analysis of bioenergetic variables in the context of both glycolysis and OXPHOS to better examine the impact of mitochondrial genome perturbations on mitochondrial function. The established patient-derived hiPSCs and their obtained differentiation derivatives such as neurons and cardiomyocytes may be used for disease modeling in vitro to improve our understanding of related early developmental deficits, the specific role of the mitochondria in patient diagnosis and prognosis and the evaluation of novel therapies for mitochondrial disorders.

### Supplementary Information

**Additional file 1**. Supplementary Figures and Tables.

## Data Availability

All the data supporting the results can be found in this manuscript and supplemental data. Please contact the corresponding author if materials generated from the current study are reasonably required.

## Abbreviations

hiPSC: Human-induced pluripotent stem cell
hESC: Human embryonic stem cell
LS: Leigh syndrome
NARP: Neuropathy, ataxia and retinitis pigmentosa
OXPHOS: Oxidative phosphorylation
mtDNA: Mitochondrial DNA
FCCP: 2-[2-[4-(Trifluoromethoxy)phenyl]hydrazinylidene]-propanedinitrile
2DG: 2-Deoxy-d-glucose
Rot: Rotenone
AA: Antimycin A
ETC: Electron transport chain
