# Supplementary material for: Induced pluripotent stem cells derived from patients carrying mitochondrial mutations exhibit altered bioenergetics and aberrant differentiation potential
Source: Stem Cell Res Ther. 2023 Nov 7;14:320. doi: 10.1186/s13287-023-03546-7 (PMC10631039; doi:10.1186/s13287-023-03546-7)
Supplement: Supplementary file 1 — Additional file 1. Supplementary Figures and Tables. [file 13287_2023_3546_MOESM1_ESM.docx]

## Supplementary Figures and Tables

**
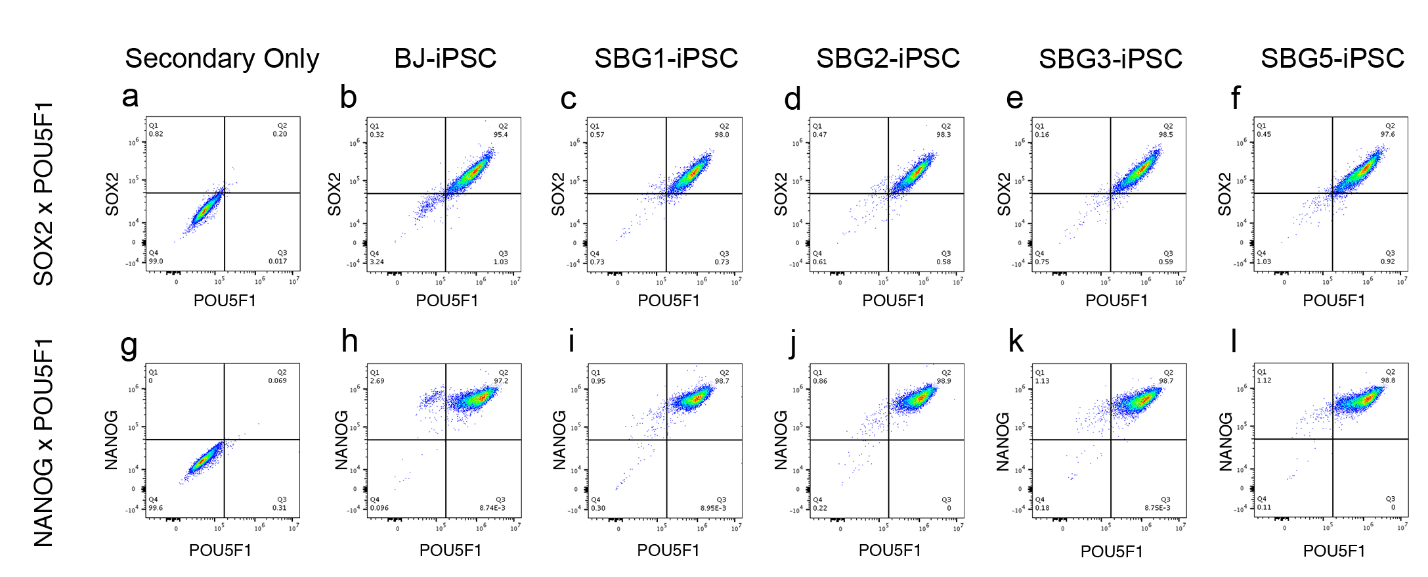
**

Supplementary Figure. 1. Mitochondrial disease patient hiPSCs co-express pluripotency markers POU5F1, SOX2, and NANOG.

Flow cytometry analysis for core pluripotency markers indicate positive co-expression of SOX2 & POU5F1 in both healthy, control BJ-iPSC (b) and diseased SBG1,2,3,5-hiPSC (c-f). Flow cytometry also confirmed co-expression of NANOG & POU5F1 in both healthy, control BJ-iPSC (h) and in diseased SBG1,2,3,5-hiPSC (i-l).


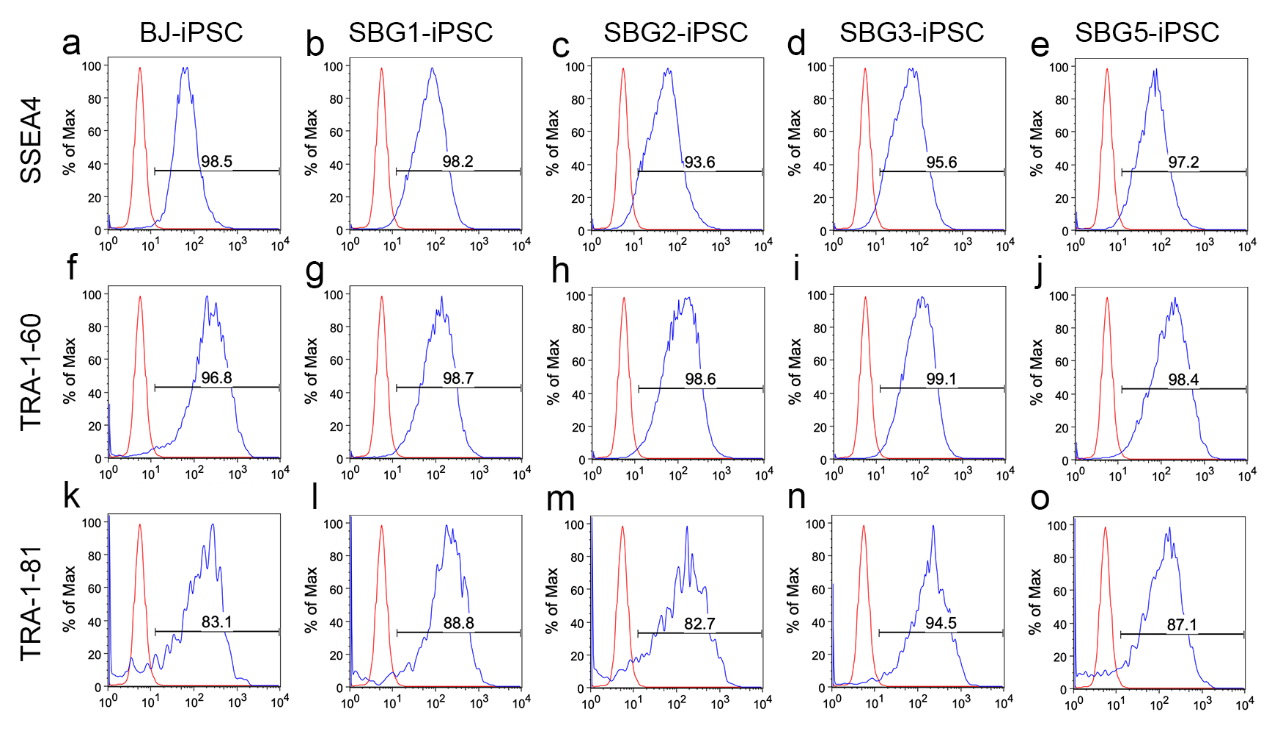


Supplementary Figure. 2. Flow cytometry shows mitochondrial diseased iPSCs express pluripotency glycoprotein and glycolipid epitopes.

Flow cytometry analysis for cell surface pluripotency markers indicate expression for SSEA4 (a-e), TRA1-60 (f-j), TRA1-81 (k-o), in both healthy, control BJ-iPSC (a,f,k) and in diseased SBG1 (b,g,l), SBG2 (c,h,m), SBG3 (d,i,n), and SBG5 (e,j,o) -hiPSC.


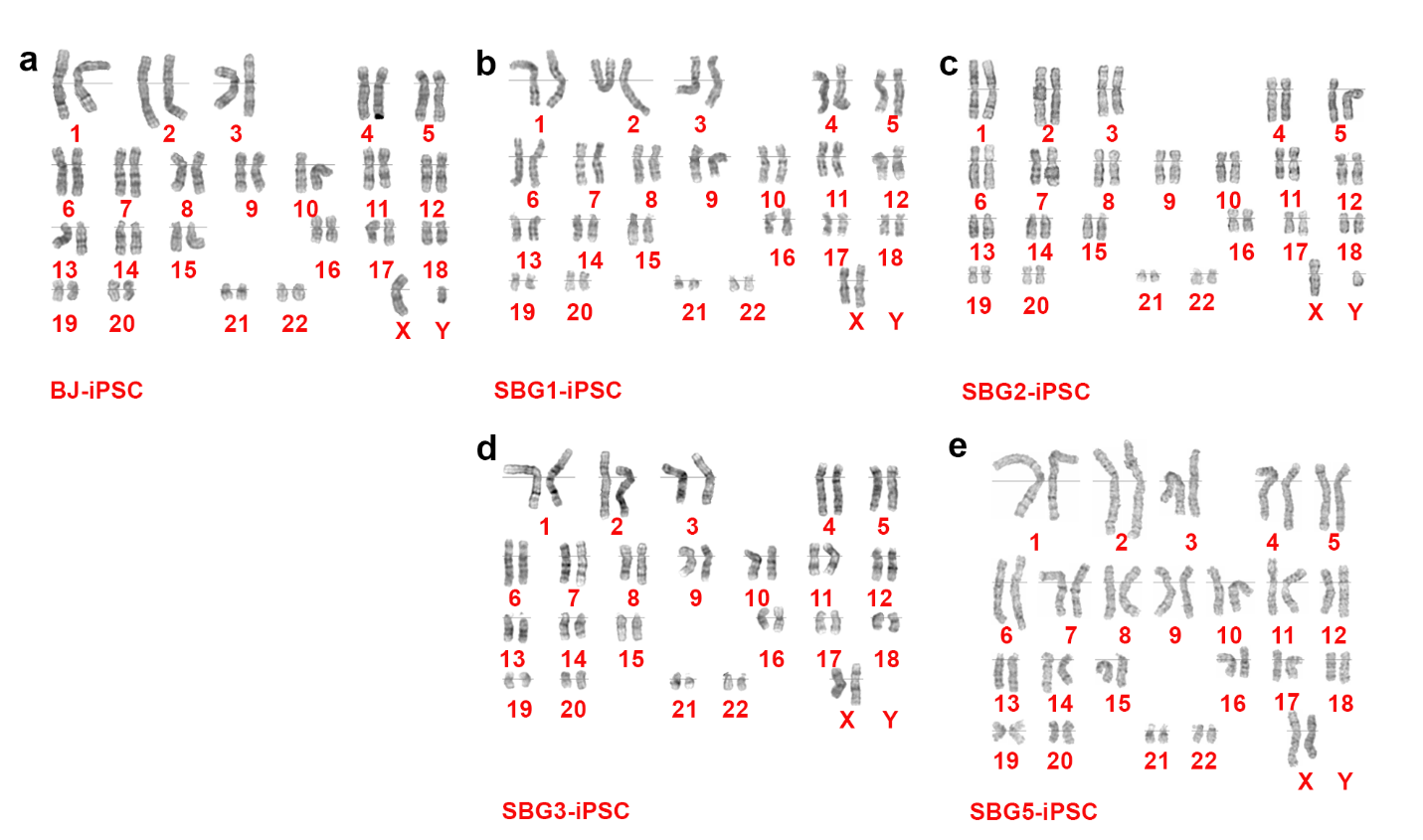


Supplementary Figure. 3. Karyotype analysis demonstrated no aneuploidies or significant DNA structural abnormalities.

Normal karyotype exhibited by (a) healthy CTL-BJ-hiPSC (46, XY); (b) *MT-ATP6*-8993T>G (SBG1-hiPSC- 46, XX); (c) *MT-ATP6*-8993T>G (SBG2-hiPSC- 46, XY); (d) *MT-ATP6*- 9185 T>C (SBG3-hiPSC- 46, XX); (e) *MTND5*-12706T>C (SBG5-iPSC- 46, XX).


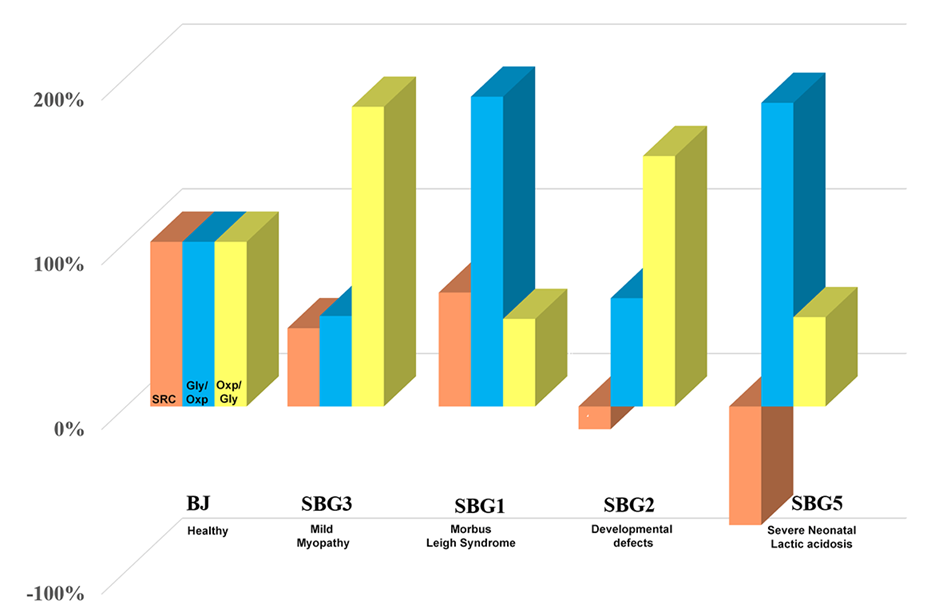


Supplementary Figure. 4. Overall bioenergetics analysis

indicates that two hiPSCs (SBG3 and SBG2) have relatively higher basal OCR rates indicative of milder phenotype, whereas two hiPSCs (SBG1 and SBG5) had lower basal OCR and higher glycolytic PER rates indicative of progression of disease. Since the mutation in SBG5 blocks proton transfer, the SRC rates are low compared to control, indicative of a very severe phenotype. The average of the data has been expressed as a percentage relative to control. Thus, the value of BJ-hiPSC is always 100%.

Supplementary Table 1. Primers for Sanger sequencing.

| **Primer Name** | **5’- 3’ Sequence** | **Product Size (bp)** |
| --- | --- | --- |
| MT-SBG1,2-F | AATGCCCTAGCCCACTTCTT | 164 |
| MT-SBG1,2-R | GCGTTTCCAATTAGGTGCAT |  |
| MT-SBG3-F | GAAATCGCTGTCGCCTTAAT | 203 |
| MT-SBG3-R | GAGGAGCGTTATGGAGTGGA |  |
| MT-SBG5-F | AAACAACCCAGCTCTCCCTAA | 220 |
| MT-SBG5-R | TCTCAGCCGATGAACAGTT |  |
